# Intestinal colonization of weaner pigs by extended-spectrum-β-lactamase-encoding *Escherichia coli* classified for differential host-association using a phylogenetics-based approach

**DOI:** 10.1038/s41598-026-53668-0

**Published:** 2026-06-10

**Authors:** M. Ferrandis-Vila, S. Mamerow, M. Bootsma, B. van der Putten, R. Oldenkamp, M. Weber, S. K. Tiwari, A. Bethe, A. Fruth, A. Fivian-Hughes, J. Leng, S. Matamoros, M. Ugarte-Ruiz, N. V. Trung, J. Alvarez, R. M. La Ragione, C. Schultsz, S. Schwarz, T. Semmler, N. T. Hoa, J. M. Ritchie, C. Menge, Christian Berens

**Affiliations:** 1 Friedrich-Loeffler-Institut, Institute of Molecular Pathogenesis, Jena, Germany; 2 Department of Mathematics, Faculty of Science, University of Utrecht, Utrecht, The Netherlands; 3 Amsterdam Institute for Global Health and Development, University of Amsterdam, Amsterdam, The Netherlands; 4 Centre for Infectious Disease Control, Institute for Public Health and the Environment (RIVM), Bilthoven, The Netherlands; 5 Amsterdam Institute for Life and Environment (A‐LIFE), Section Chemistry for Environment and Health, Faculty of Science, Vrije Universiteit Amsterdam, Amsterdam, The Netherlands; 6 Robert Koch Institute, Genome Competence Centre (MF1), Berlin, Germany; 7 Food, Microbiome, and Health Research Programme, Quadram Institute Bioscience, Norwich, UK; 8 Institute of Microbiology and Epizootics, School of Veterinary Medicine, Freie Universität Berlin, Berlin, Germany; 9 German Environment Agency (UBA), Berlin, Germany; 10 Department for Infectious Diseases, Division of Enteropathogenic Bacteria and Legionella (FG11), National Reference Centre for Salmonella and other Enteric Bacterial Pathogens, Robert Koch Institute, Wernigerode, Germany; 11 School of Biosciences, Faculty of Health and Medical Sciences, University of Surrey, Guildford, UK; 12 School of Veterinary Medicine, Faculty of Health and Medical Sciences, University of Surrey, Guildford, UK; 13 Department of Medical Microbiology and Infection Prevention, Amsterdam UMC, University of Amsterdam, Amsterdam, The Netherlands; 14 VISAVET Health Surveillance Center, Universidad Complutense, Madrid, Spain; 15 Oxford University Clinical Research Unit, Ho Chi Minh City, Vietnam; 16 Department of Animal Health, Faculty of Veterinary Sciences, Universidad Complutense, Madrid, Spain; 17 Veterinary Centre of Resistance Research (TZR), School of Veterinary Medicine, Freie Universität Berlin, Berlin, Germany; 18 Centre for Tropical Medicine and Global Health, Nuffield Department of Medicine, University of Oxford, Oxford, UK; 19 Biomedical Research Center, Pham Ngoc Thach University of Medicine, Ho Chi Minh City, Vietnam; 20 Friedrich-Loeffler-Institut, Institute of Molecular Pathogenesis, Naumburger Str. 96a, D-07743 Jena, Germany

**Keywords:** Colonization, Host specificity, Phylogenetic classification, *Escherichia coli*, Pig, Isolate competition, Extended-spectrum beta-lactamase, Microbiology, Molecular biology

## Abstract

Intestinal bacteria in healthy humans and animals are important reservoirs of antimicrobial resistance (AMR) genes. Certain *Escherichia coli* lineages dominate the global spread of AMR, but the extent of their host specificity and its impact on transmission remains unclear. Here, 1,198 *E. coli* isolates from cattle, chickens, humans, and pigs across Germany, the UK, Spain, and Vietnam were classified for host-association using a phylogeny-based bioinformatic approach, with non-host-associated isolates defined as ‘generalists’. To validate this classification, 17 extended-spectrum beta-lactamase (ESBL)-producing isolates representing host-associated or generalist clusters were administered as a cocktail to three groups of weaner pigs, either untreated or treated with amoxicillin or ceftiofur. Bacterial shedding was monitored via culture and isolate-specific PCR up to 56 days post-inoculation. Colonization occurred within 24 h, with shedding peaking early and declining over time. Only 12 of 17 isolates were recovered post-inoculation; four were persistently detected, including one pig-associated, one bovine, one chicken, and one generalist isolate. These findings suggest limitations in the classification approach, possible low host selectivity in pigs, and/or incomplete knowledge of colonization mechanisms. Nonetheless, the model successfully identified isolates capable of stable colonization, supporting its utility for studying AMR *E. coli* host adaptation.

**Supplementary Information:**

The online version contains supplementary material available at10.1038/s41598-026-53668-0.

## Introduction

Bacteria live and proliferate in many different habitats and hosts, and under various conditions^1^. Each niche provides specific conditions that allow certain bacteria to replicate, while others do not^2^. Based on their ability to populate biotic and abiotic niches, bacteria are either broadly classified as generalists, associated with many different hosts and living environments, or as specialists, restricted to one or a small range of hosts or niches^3^. Host-bacteria associations can result in a symbiotic outcome, but mutualistic, negative pathological or antagonistic interactions are also possible^4^.

Understanding these bi-directional relationships requires the study of multiple factors, including genes and gene groups that contribute to the colonization of a specific host. Next generation whole genome sequencing (WGS), a powerful, rapid and sensitive tool, provides the basis for a detailed understanding of bacterial populations and adaptive evolutionary changes therein^5^, and has been an instrumental aid in identifying host-adaptive genetic markers. Interpretation of next generation WGS data is often supported by phenotypic studies to confirm which genes and mechanisms are involved in host specificity^2,6^. Such experimental studies are needed to determine which genetic determinants contribute to host-specificity and which biological mechanisms are functionally involved in successful and sustained colonization of specific host species.

In experimental microbiology, the colonization ability of isolates and their impact on the host are frequently assessed by inoculating animals with selected bacterial isolates or with a mutagenized pool derived from a single isolate as in the signature-tagged mutagenesis approach^7–10^. However, this approach is laborious, time-consuming and expensive, as only a single isolate or an isolate-derived mutant pool is evaluated per animal group at a time. Multiple-isolate or ‘cocktail’ inoculations, on the other hand, offer the possibility to analyze several isolates in parallel, yet require a more demanding study design and read-out and are, thus, less frequently used^11^. A major obstacle is the reliable identification of the individual inoculated isolates post-challenge, since isolates from the same bacterial species present in the resident microbiome may have similar phenotypic and genotypic profiles.

*Escherichia coli* is an important bacterium in the microbiota of animals and humans. It is extremely well-studied^12–14^, and one of the most prevalent Gram-negative facultative anaerobes in the human and animal gastro-intestinal tracts^15,16^. *E. coli* displays great phylogenetic diversity, allowing it to adapt to a large variety of hosts and to display different types of host interaction, varying from a mainly mutualistic, commensal lifestyle to overtly pathogenic lifestyles due to the acquisition of different sets of virulence genes^17–21^. Isolate phenotypes also vary among non-pathogenic commensal lineages, ranging from safeguarding against colonizing pathogens to aggravating inflammation, which can be induced by a variety of stimuli^19,22^. Beyond this dichotomy, colonization by antimicrobial resistant bacteria is of particular concern to public and veterinary health because of the potential transfer of antimicrobial resistance (AMR) genes to other bacterial species^23^. This particularly holds true for *E. coli*, with its ubiquitous distribution and genomic plasticity. Indeed, variable phenotypic resistance and virulence gene configurations have been observed in different *E. coli* lineages, demonstrating that the commensal flora is a potential reservoir of virulence and AMR determinants^24^. Colonization of the gut by AMR bacteria results from the bi-directional relationship between members of bacterial communities and the host but is also facilitated by selection-based adaptation to antimicrobial treatment^23^.

Achieving a better knowledge of *E. coli* host adaptation is particularly challenging due to the inherent complexity of the species’ population structure. However, this is of prime importance for understanding isolate maintenance and spread, as well as resistance gene uptake, reshuffling and dissemination, thereby contributing to our ability to elaborate risk assessments of the epizootic and zoonotic potential of individual *E. coli* isolates.

Herein, we applied a phylogeny-based classification methodology to assign host specificity scores to a large collection of *E. coli* obtained from healthy and diseased hosts (cattle, chickens, humans and swine) across four geographic locations (Germany, Spain, the UK, and Vietnam)^6^. While the collection also included isolates from wild boars, these were not used to define host-associated groups due to low sample numbers and limited representation relative to the main host categories; nonetheless, wild boars are relevant at the livestock–wildlife interface and may contribute to the exchange of enteric bacteria and AMR determinants with domestic animals^25^. In addition, the isolate collection and, consequently, the subset eligible for experimental inclusion, was dominated by isolates originating from Germany, which limits geographic generalization of host-association patterns.

To evaluate whether phylogeny-based host-association can inform on in vivo colonization behaviour, we selected a subgroup of seventeen extended-spectrum β-lactamase (ESBL)-producing isolates representing host-associated and ‘generalist’ clusters and experimentally determined their ability to colonize pigs. ESBL-producing *E. coli* were prioritized because of their relevance in human and veterinary medicine. These lineages represent important reservoirs of transferable resistance determinants; however, their frequent occurrence across multiple hosts may indicate a reduced host restriction relative to non-ESBL isolates. Pigs were chosen as experimental model due to their global economic significance and their role as a reservoir of epizootic and zoonotic microorganisms, including antimicrobial resistant *E. coli*^26^. The latter is due to the anatomical and physiological similarities between the swine and human gastrointestinal tracts as well as dietary similarities, which also contribute to pigs and humans having similar enteric microbiota^27^. The isolates were administered intra-gastrically as a single composite inoculum (“cocktail”) and colonization was monitored for up to 56 days post-inoculation (dpi), with or without exposure to selective or co-selective antimicrobial agents. Overall, this study aimed to experimentally assess how well a phylogeny-based host-association framework aligns with colonization outcomes in pigs, and to identify isolates capable of stable colonization under different antimicrobial exposures.

## Results

### Host-association classification of the *E. coli* isolates from the collection

**Fig. 1:**
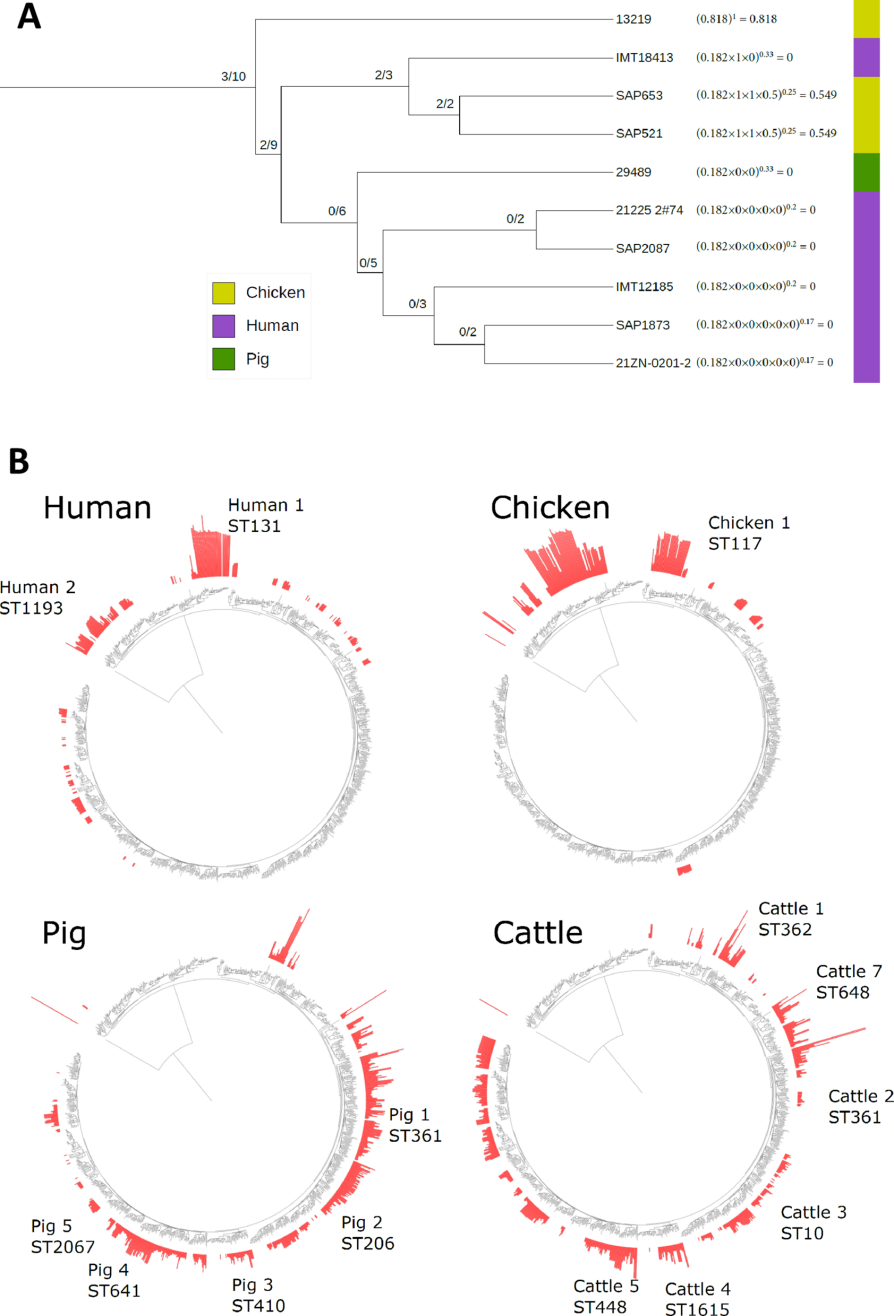
Host specificity scores for all isolates from the *E. coli* collection. (**A**) Example of calculating the host specificity score for association with the chicken host along a small branch of a single phylogeny tree (in detail in the Supplemental Material). The higher the resulting number, the higher the likelihood of specific adaptation to chickens. (**B**) The specificity score of each isolate was calculated for each of the four hosts and plotted on the pangenome phylogeny map of all 1,198 isolates. Scores are shown in red for the hosts: Human, Chicken, Pig and Cattle. For each host, isolate clusters identified are numbered and shown with their major sequence type (ST; Achtman scheme^28^. For a closeup of the Pig3/Cattle4 cluster, see Supplemental Figure S1.

The genomes of 1,198 *E. coli* originally collected from healthy and diseased cattle, chickens, humans, swine and wild boars in Germany, Spain, the United Kingdom and Vietnam between 2003 and 2018 were included in the phylogeny-based analysis^6^. For each isolate, a host score was calculated from the pangenome phylogeny and the host origin of neighbouring isolates (Fig. 1A) yielding values between 0 and 1 that reflect the degree of phylogenetic clustering with isolates from a given host. Full details can be found in Materials and Methods.

Visualisation of the host specificity scores for isolates that met the main selection criteria revealed 14 host-specific groups that were associated with humans (human 1 and 2), chickens (chicken 1), pigs (pig 1 to 5) and cattle (cattle 1 to 7) (Fig. 1B). Groups of isolates containing high scores for humans and chickens were closely localised within the phylogeny, whereas those for cattle and pigs were more broadly distributed. Each group comprised of only one or two sequence types, indicating genetic relatedness within the groups. Four groups were designated as containing ‘generalists’; isolates within these groups yielded host specificity scores above zero for two or more host species. For inclusion into the generalist groups, where host specificity scores for the individual isolates were available across multiple host species, the geometric mean of the host specificity score ranks was calculated, with lower geometric means indicating a stronger association with multiple hosts. Wild boar isolates were included in the underlying phylogeny but were not assigned to a host-associated group because their number and phylogenetic representation were insufficient to define robust host-associated clusters under the criteria applied (see Materials and Methods).

Host-associated clusters were not assigned to isolates that did not meet the additional inclusion criteria, even when their host-association score was high. Notably, many isolates were excluded at this stage because they did not carry an ESBL gene.

Due to the combined selection constraints (multi-host score threshold, ESBL genotype, and virulence factor filtering), the number of isolates eligible for inclusion as generalists was limited. This was most evident for the generalist_2 group, which overlapped phylogenetically with cattle-associated isolates from group 4 (see the supplemental material).

### Isolate selection for animal experiments

After applying the additional inclusion and exclusion criteria (see Materials and Methods), 84 eligible isolates exhibiting an ESBL genotype were compiled. This list was reduced to 27 isolates (Table S1) based on (i) preference for isolates from clinically healthy hosts to minimize the risk of disease in experimental animals, (ii) a lack of known toxins (Shiga toxin, heat-stable and labile enterotoxins), and (iii) a low number of other known *E. coli* virulence factors.

The remaining isolates were subjected to preliminary screening to assess antimicrobial susceptibility profiles, acid resistance, and colicin production (procedures and criteria are described in the supplemental material). Six isolates were excluded because they were colicin-positive. From the remaining 21 isolates, one isolate per host and per cluster was chosen, to have the same number of bacterial isolates per group while reducing the isolate number to simplify detection via multiplex-PCR^29^. From three clusters, not designated in Fig. 1, no isolate was selected due to (i) the presence of Shiga toxin-encoding genes in group Cattle6 and (ii) the high total number of virulence factor-encoding genes (*n* = 97) found in the single isolate present in cluster Human3. Of note, this isolate contained 23 more virulence factors than the neighbouring isolate and 54 more than the mean number of virulence factors per isolate in the final selection (mean ± standard deviation: 43 ± 15). Seventeen isolates (Table 1, S2-S4) were ultimately selected for experimental infection, including one isolate, that was classified into two different groups (Generalist2 and Cattle4), because it met the host-association category criteria under each of the different classification frameworks applied (see Materials and Methods and Supplemental Figure S1). It was ultimately included as Cattle4 (Table 1). All isolates were made rifampicin-resistant to facilitate their retrieval (see Materials and Methods).

**Table 1:** *E. coli* isolates selected for use in this study.

Isolate ID	Host	Cluster	Country of origin	Host health status*	Year of isolation	Phylogroup	Multi-locus sequence types (ST, Achtmann^28^)
21225_2#112	Chicken	1	Vietnam	Healthy	2013	F	ST1163
SAP1847	Human	1	UK	UTI	2017	B2	ST131
SAP1710	Human	2	UK	UTI	2017	B2	ST636
IMT38565	Cattle	1	Germany	Diarrhea	2016	D	ST362
R45	Cattle	2	Germany	Healthy	2015	A	ST361
IMT13936	Cattle	3	Germany	Mastitis	2007	A	ST10
IMT34414	Cattle	4	Germany	Diarrhea	2014	C	ST88
IMT10909	Cattle	5	Germany	Salmonellosis	2005	B1	ST448
9475_4#43	Cattle	7	Germany	Healthy	2011	F	ST648
IMT39234	Pig	1	Germany	Diarrhea	2016	A	ST361
IMT28138	Pig	2	Germany	Enteritis	2011	A	ST206
39533	Pig	3	Germany	Healthy	2011	C	ST410
IMT38723	Pig	4	Germany	Healthy	2016	B1	ST641
IMT38701	Pig	5	Germany	Healthy	2016	B1	ST2067
21225_2#178	Generalist	1	Vietnam	Healthy	2013	B1	ST162
09–05726	Generalist	3	Germany	Diarrhea	2009	A	-
ZTA1601993EC	Generalist	4	Spain	Healthy	2016	D	ST1011

- = not belonging to a defined sequence type.

*Clinical information was derived from the accompanying sample metadata.

### Shedding of *E. coli* isolates following experimental inoculation of weaner pigs

**Fig. 2:**
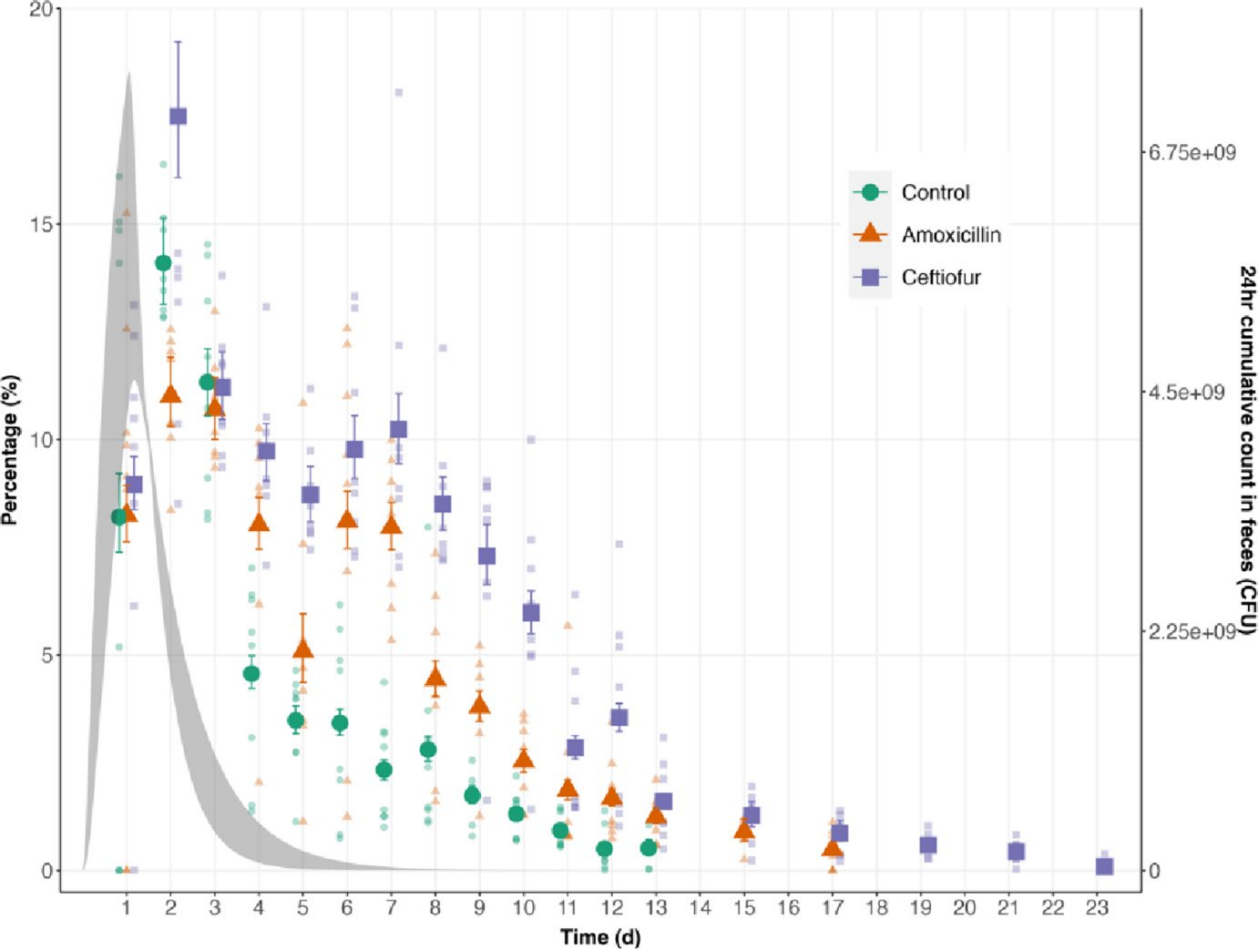
Percentage of experimental isolates over total colony counts (left y-axis) and modelled 24-hour cumulative counts of inert particles in feces (right y-axis). Measured data are shown as coloured symbols: large filled symbols represent the most likely aggregate values per treatment group and time point, with error bars indicating 95% confidence intervals; smaller symbols represent estimates for individual pigs. The grey shaded polygon depicts the model output: a fusion of multiple simulations of inert particle transit through the gastrointestinal tract of piglets following an inoculum of 10¹⁰ particles. This output aggregates different realistic model settings (as detailed in Figure S2). Note that the two y-axes use different scales and units; hence, comparisons should focus on temporal dynamics (x-axis) rather than on vertical alignment.

To investigate the colonisation ability of the *E. coli* isolates, eight- to nine-week-old weaner pigs were experimentally inoculated with 10^10^ CFU of the 17-member *E. coli* cocktail and the presence of *E. coli* in rectal content was monitored over time. Fecal shedding of experimental isolates, i.e., growth of double antimicrobial-resistant *E. coli* (ceftiofur and rifampicin (CET + RIF) or amoxicillin and rifampicin (AMX + RIF)) relative to growth of enterobacteria, peaked at 2 dpi with the animals treated with ceftiofur (CET) exhibiting a higher percent recovery than those treated with amoxicillin (AMX) or left untreated (control (CON)) (Fig. 2).

By 4 dpi, percent recovery from the CON group had declined and values from all groups significantly differed from each other (CON vs. AMX, *p* = 0.0019; CON vs. CET, *p* = 0.0002; AMX vs. CET, *p* = 0.0003) until 7 dpi. Between 8 and 12 dpi, percent recovery in rectal content was significantly greater in the CET group compared to those from animals in the CON (*p* = 0.02) and AMX groups (*p* = 0.02). On 13 dpi, the CET group shed significantly higher mean relative CFU numbers than that of the AMX group, while the absolute CFU counts for the CON group dropped below the detection limit, prompting the introduction of an enrichment step in the sample processing protocol to assess strain presence. Enrichment steps were introduced for the AMX group at 17 dpi and for the CET group at 23 dpi. Following the introduction of enrichment, colony counts were no longer used for quantitative comparisons. The uncertainty in the coliform counts and ratios was assessed by modelling the sampling process, resulting in narrow 95% confidence intervals and indicating consistent shedding dynamics among pigs within each treatment group (Fig. 2). Only a small number of time points showed appreciable deviations at the individual animal level.

The model revealed that the peak of RIF-resistant isolates as percentage of the total coliform count (symbols on Fig. 2) occurred approximately one day later than the simulated passage of inert particles through the pig digestive tract (shadowed polygons on Fig. 2; Supplemental Figure S2). This delay compared to passage of non-interacting inert particles, as well as the detection of experimental bacteria at numbers above the simulation curve, can be interpreted as resulting from colonization, replication and delayed release from the pig intestinal tract.

### Influence of antimicrobial treatment on *E. coli* shedding patterns

**Fig. 3:**
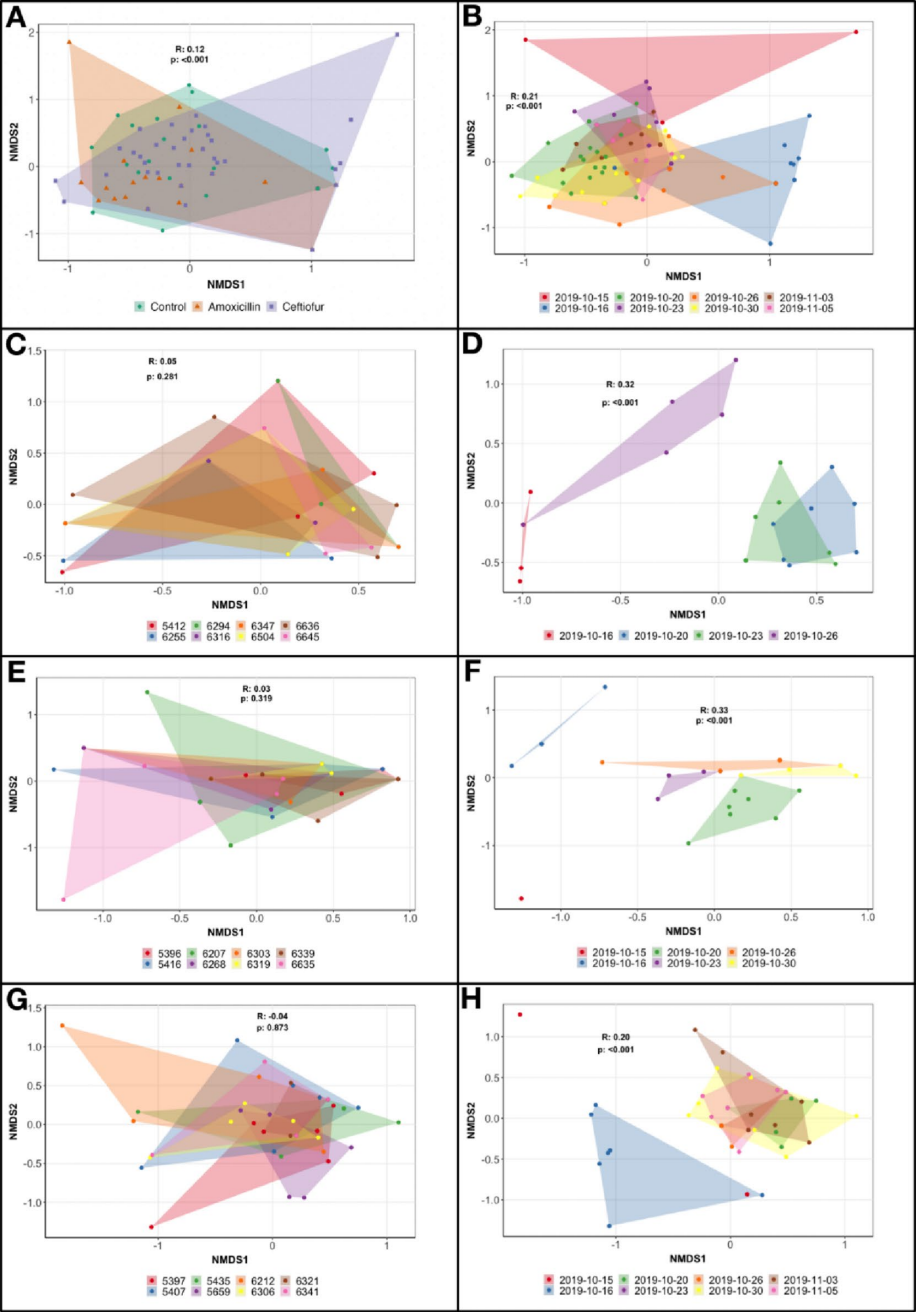
(Dis)similarity of the qualitative experimental *E. coli* isolates’ shedding patterns of pigs along the first two NMDS-dimensions. (**A**) between treatment groups (regardless of sampling time point); (**B**) between time points (regardless of treatment group). (**C**)-(**H**) for the individual pigs in the different treatment groups. (**C**, **D**): control group, (**E**, **F**): amoxicillin treatment group, (**G**,** H**): ceftiofur treatment group. Graphs in the left column: comparisons between pigs (regardless of sampling time point); right columns: comparison between sampling time points (regardless of pig). Each symbol represents a unique profile sampled from a single pig. Shaded polygons represent the convex hull of all profiles belonging to the specific set of profiles. R: ANOSIM R with p value.

To compare *E. coli* strain shedding across the different animals, non-metric multidimensional scaling (NMDS) analysis was performed. The resulting profiles were more similar within each treatment group than between the treatment groups (Fig. 3A; ANOSIM *R* = 0.12, *p* < 0.001). The profiles were also more similar at each sampling time point than between the different time points (Fig. 3B; ANOSIM *R* = 0.21, *p* < 0.001). indicating that time had a stronger effect on isolate shedding than antimicrobial treatment. This was also detected within each treatment group, as samples from different pigs at a single time point were more similar than samples from single pigs at different time points. This holds true for all three treatment groups (Fig. 3C- H), suggesting that the shedding patterns by pigs receiving the same treatment converged over time.

### Qualitative shedding patterns of individual *E. coli* present in the cocktail

One animal from the CET group was already PCR-positive for 13 of the 17 experimental isolates in the first rectal sample collected at hour 6 pi, showing successful passage of the cocktail rapidly through the intestine (data not shown). On average, the most diverse *E. coli* population was found on 5 dpi, with 12 out of 17 isolates recovered (Figure S3). Thereafter, colonization diversity decreased to 7–8 isolates shed on single days, on average. After enrichment, the number of isolates detected in the samples increased again transiently (Figure S4), and, with an average of 8–9 isolates, remained slightly higher compared to the diversity detected before enrichment.

**Fig. 4:**
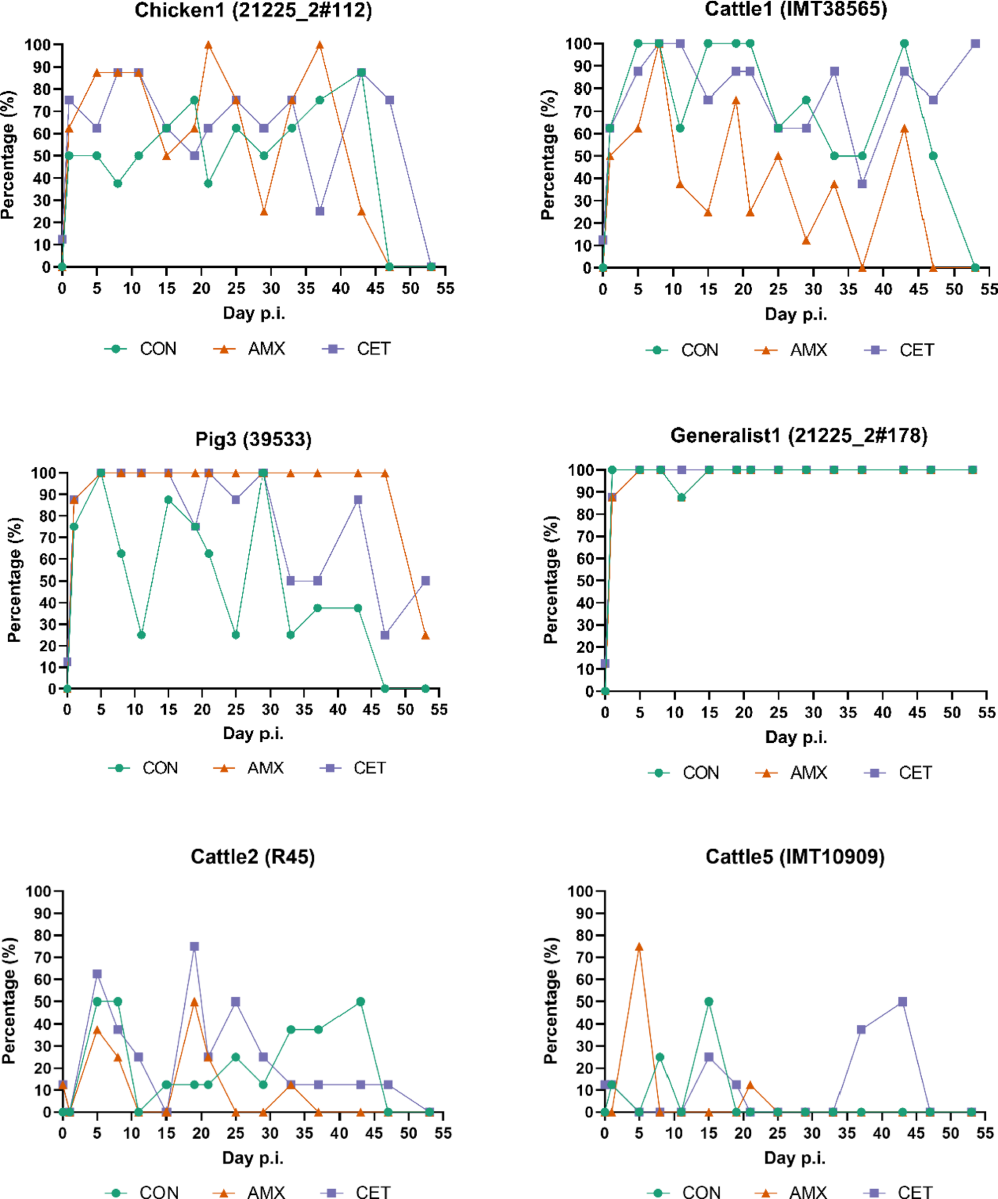

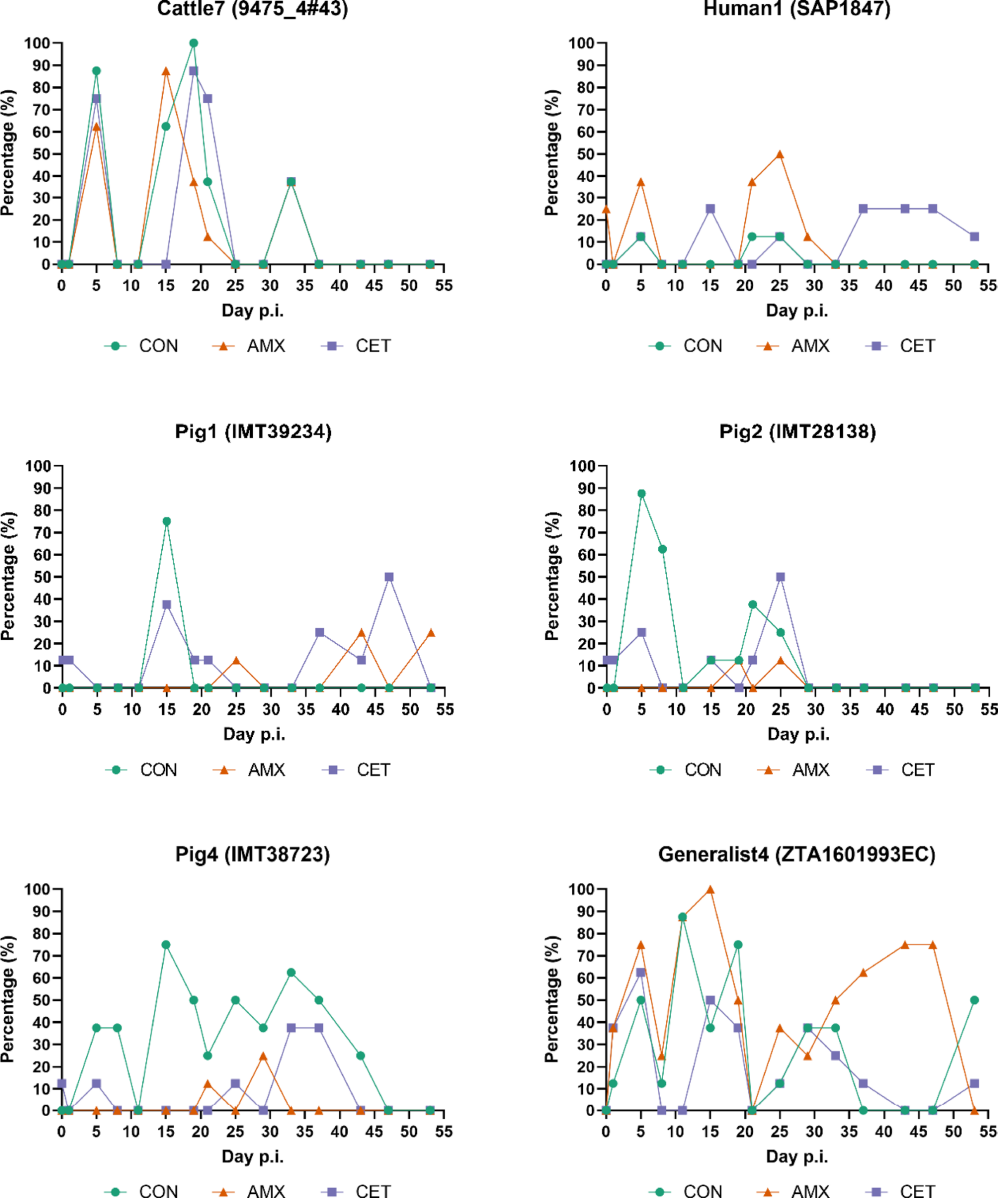
Percentage of weaner (later juvenile) pigs shedding specific *E. coli* isolates administered as a single inoculum (‘cocktail’). Pigs were intra-gastrically inoculated with a 17-member *E. coli* cocktail and the presence of individual isolates monitored in rectal content over time. Weaners were exposed to saline (CON, control), amoxicillin (AMX) or ceftiofur (CET) the day before, the day of, and the day after inoculation. Fecal *E. coli* were recovered on antibiotic selection plates and a multiplex PCR-based detection approach was used to identify the presence/absence of individual members. The X axis shows days post-inoculation (p.i.), the Y axis shows the percentage of animals positive for a specific isolate. *N* = 8 animals per group.

Regarding colonization of the pigs by specific isolates, we detected different presence/absence patterns for different isolates. Four isolates were consistently present in most animals from the beginning to the end of the experiment, namely Chicken1 (21225_2#112), Cattle1 (IMT38565), Pig3 (39533) and Generalist1 (21225_2#178), with Generalist1 being the most dominant isolate, followed by Pig3 (Fig. 4). Notably, Generalist1 was consistently shed by all animals, regardless of antimicrobial treatment, starting from 12 h post-inoculation. Surprisingly, isolates, such as Chicken1 and Cattle1, categorized as having a stronger host preference for other species, were also detected in the rectal content of most animals in all treatment groups. Of the remaining eight experimental isolates, Cattle2 (R45), Cattle5 (IMT10909), Cattle7 (9475_4#43), Human1 (SAP1847), Pig1 (IMT39234), Pig2 (IMT28138), Pig4 (IMT38723), and Generalist4 (ZTA1601993EC) were shed by only a subset of animals at each time point, independent of treatment group. This was particularly evident for isolates Pig1 and Pig2, which exhibited only occasional detectability (Fig. 4).

Five isolates (Human2 (SAP1710), Cattle3 (IMT13936), Cattle4 (IMT34414), Pig5 (IMT38701) and Generalist3 (09–05726)) were not detected, or if detected, the representation was so minimal—limited to a single timepoint or two in only one individual—that it was deemed inconsequential. This unexpected outcome was noteworthy for the isolate designated as Generalist3, in addition to the one classified as specific for the animal species (Pig5) used in this model.

### Phylogeny-based classification method and colonization capacity

To assess the performance of the classification method in predicting an isolate’s colonization capacity, the area under the curve (AUC) was calculated from the shedding pattern of each specific isolate (Fig. 4) from 4 to 13 dpi. This interval was selected to represent the period after initial transit and before the introduction of enrichment in the first group; AUC calculations were restricted to time points where quantitative comparisons of CFU ratios were still valid (see Materials and Methods and Table S5).

**Fig. 5:**
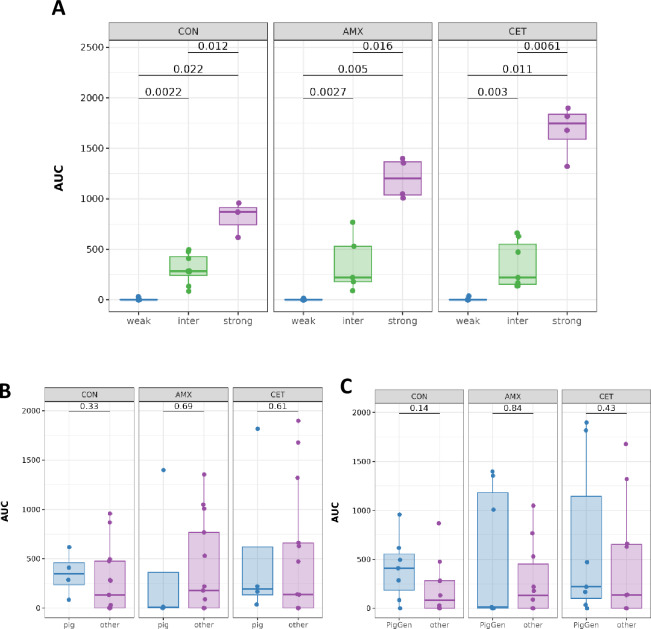
Comparison of colonization behaviour of the inoculated isolates. Shown is AUC data as individual data points (coloured dots) in a Box-Whisker-Plot representation for isolates classified either as weak (blue), intermediate (green) or strong (violet) colonizers (**A**), or as associated with the pig (blue) or another and no (violet) host (**B**), or as associated with the pig and no host (‘generalist’; blue) or with the other hosts (violet) (**C**). Significant (*p* < 0.05) or non-significant (*p* ≥ 0.05) differences in their mean AUC values are displayed above the corresponding groups. Whiskers englobe all values within 1.5xIQR.

The AUC values were used to classify isolates as strong, intermediate, or weak colonizers (see Materials and Methods and Table S5). Mean AUC values significantly differed between colonizer classes for each treatment group (Fig. 5A). Strong colonizers reached highest mean AUC values in the CET treatment group and lowest in the CON group, whereas values for intermediate and weak colonizers were comparable between treatment groups.

To assess the correlation between in silico predicted host specificity and in vivo colonization ability, AUC values of pig-associated isolates were compared to those associated with other hosts (Fig. 5B). Additionally, pig-associated and generalist isolates were compared collectively against cattle-, chicken-, and human-associated isolates (Fig. 5C). Neither comparison yielded significant differences, indicating that host-association inferred from phylogeny and isolation host did not predict colonization capacity in pigs in this experiment. Colonization patterns varied between treatment groups, with the exception of Generalist1 and Pig3, which consistently showed strong colonization across groups.

### *E. coli* presence and diversity within the intestinal tract

*Post-mortem* analysis of intestinal tissues and contents confirmed the presence of bacterial colonization beyond what was observed in rectal samples. Strong colonizers (Generalist1, Pig3, etc.) were consistently detected across all treatment groups, validating their classification, though some variability was observed (e.g., Pig3 was often absent in control samples despite fecal shedding patterns) (Table 2A-B).

Sample enrichment significantly increased the detection of isolates, uncovering a larger diversity, including intermediate and weak colonizers, that were not always identifiable in non-enriched samples. This was particularly notable in the CET group, which showed the highest strain diversity post-enrichment (Table 2B).

Also, strain detection varied between tissue and content samples taken from the same intestinal locations, indicating that colonization was not uniformly distributed across gut compartments. While many isolates appeared in both sample types, some were exclusive to either tissue or content, underscoring localized differences in colonization dynamics (Table 2A-B).

**Table 2:**
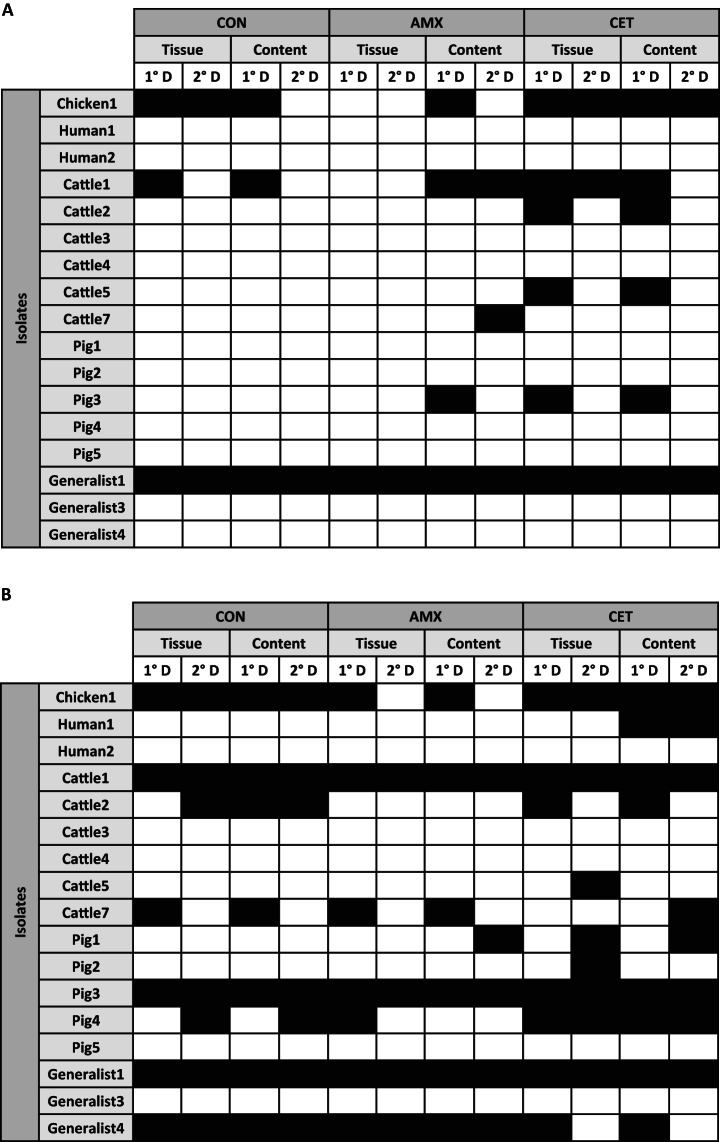
Summary of positive PCR signals of the experimentally inoculated isolates in the different animal groups with or without sample enrichment, for each dissection timepoint. Table 2A displays results from non-enriched samples after collection. Table 2B displays results from samples after undergoing an additional enrichment step to favour experimental isolate growth. Black boxes denote the presence of a specific isolate in at least a single individual animal per group and dissection day, while empty boxes represent lack of presence in all animals of a group and dissection day (*n* = 4 animals per group and dissection day).

1°D: First *post-mortem* examination 43 dpi.; 2°D: Second *post-mortem* examination 56 dpi.

### Quantitative colonization

From the 17 isolates included in the inoculation cocktail, only four were selected for qPCR analysis, as these were the only isolates that could be recovered directly from the final rectal swabs prior to sacrifice without selective enrichment. As a result, qPCR analyses focused on isolates with robust detectability at the end of the experiment, and did not provide quantitative resolution for isolates that were detected only sporadically or only after enrichment.

**Fig. 6:**
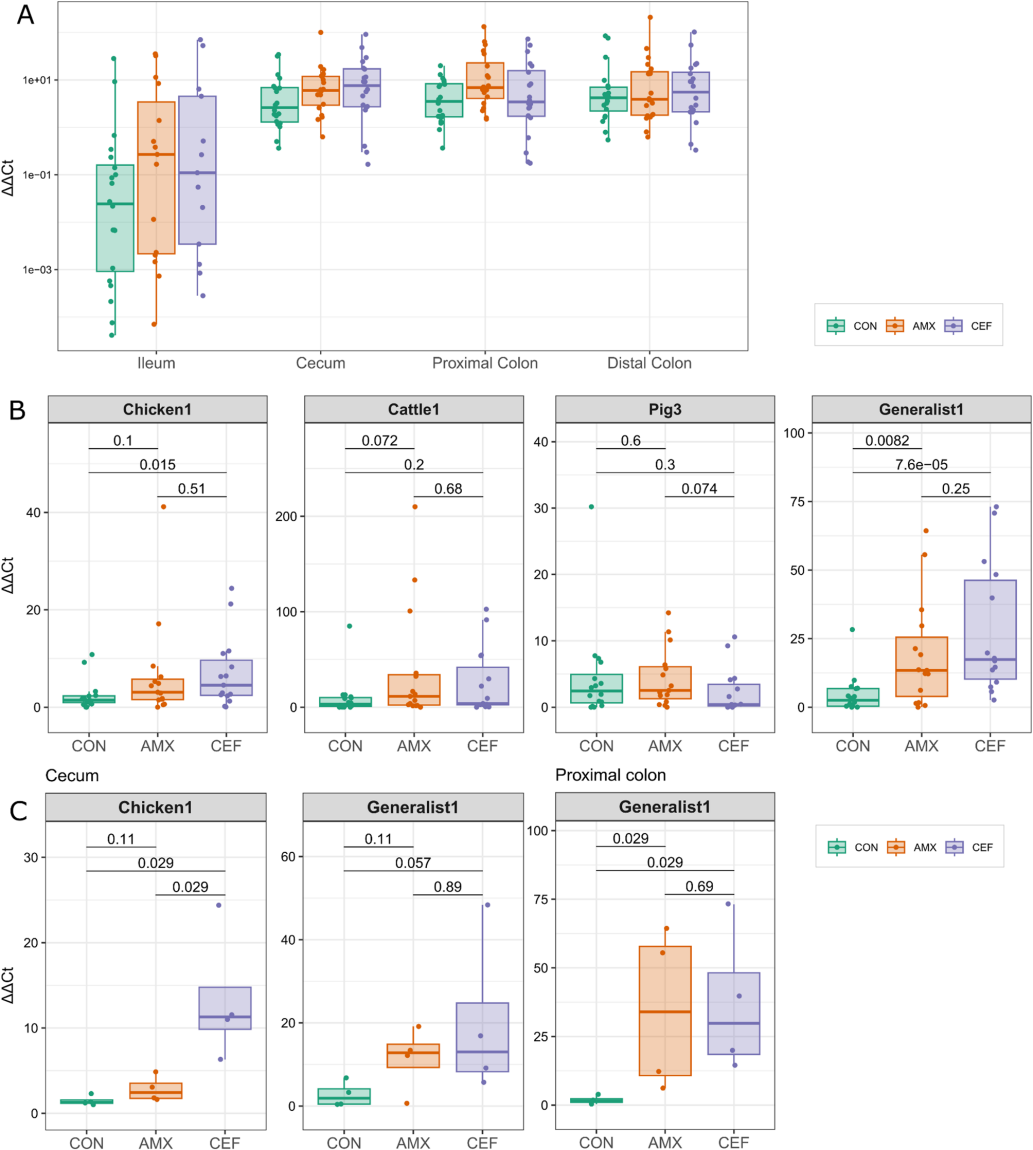
Quantitative assessment of experimental *E. coli* isolate colonisation in intestinal compartments by qPCR. (**A**) Comparison of the presence of experimental isolates in the content of the different intestinal areas at both dissection dates (pooled data). Shown is qPCR data as individual data points (coloured dots) in a Box-Whisker-Plot representation for the three treatment groups in the different intestinal locations. (**B**) Comparison of presence of strong colonizing experimental isolates in the overall intestine *post-mortem*. (**C**) Comparison of presence of strong colonizing experimental isolates in cecum and proximal colon *post-mortem*. Green – CON, orange – AMX, purple – CET. Whiskers englobe all values within 1.5xIQR.

The qPCR results showed few significant differences between the different treatment groups. With respect to the presence of isolates in the *post-mortem* content samples, the ileum showed the most disperse distribution of values, while cecum, proximal and distal colon showed very similar distributions (Fig. 6A).

When treating the entire intestine as a single organ (merging all intestinal sites together), a few significant differences in colonization were observed. Chicken1 colonized significantly better in the CET group vs. the CON group (*p* = 0.015, p.adj = 0.046) and Generalist1 displayed significantly better colonization in both treatment groups (AMX, *p* = 0.0082, p.adj = 0.012 and CET, *p* = 7.6e-05, p.adj = 2.3e-04) compared to the CON group. (Fig. 6B).

When looking at specific intestinal locations, the only significant differences were observed in the cecum for Chicken1, between the AMX and CET (*p* = 0.029, p.adj = 0.043) and the CON and CET (*p* = 0.029, p.adj = 0.043) groups, and for Generalist1 in the proximal colon between both treatment groups (AMX, *p* = 0.029, pad.j = 0.043 and CET, *p* = 0.029, p.adj = 0.043) and the CON group (Fig. 6C).

## Discussion

Bacterial host specificity refers to the ability of bacteria to selectively colonize different host species^30^, with many bacteria exhibiting broad host ranges, spanning from insects to mammals, and some having narrow host ranges, encompassing only one or a few species. However, the outcome of such a colonization event is not only influenced by bacterial genetics but also by host factors, with some bacteria being innocuous or even beneficial for some hosts, while being pathogenic for others^31^. Therefore, understanding the physiological and genetic basis underlying bacterial host specificity is of prime importance, potentially providing novel targets for therapeutic control of bacterial diseases^32^. This study employed an in vivo weaner/juvenile pig model to investigate gastrointestinal colonization by a defined cocktail of ESBL-encoding *E. coli* isolates, classified by a phylogeny-based host-association framework. In the animal model, the experimental isolates encounter a wide variety of cell types, from both microbiota and host, including immune cells, and they are exposed to the normal physiological processes of the digestive tract – a complexity that cannot yet be fully replicated under in vitro conditions.

Considering colonization as the presence of an isolate for longer than three times the duration of inert particles passing through the animal’s gastrointestinal tract^33^, we observed colonization by several of the inoculated isolates in all three treatment groups, albeit to different extents. Notably, colonization was most pronounced in animals treated with ceftiofur and least in those from the control group, correlating with the ability of the respective treatment to disrupt the host microbiota, a major factor in mediating colonization resistance^34–36^. In Germany, cephalosporin use in pigs is relatively limited compared with more commonly used beta-lactam ring containing compounds, such as aminopenicillins^37,38^, and ceftiofur is classified by the Antimicrobial Advice ad hoc Expert Group (AMEG) as Category B (“Restrict”), reflecting stewardship concerns. European surveillance data from the period surrounding our animal experiment indicate that 3rd - and 4th -generation cephalosporins accounted for only a small proportion of total veterinary antimicrobial sales in most reporting countries^40^. Nevertheless, stewardship policies and sector-specific practices vary; for example, the UK pig sector reported discontinuation of routine 3rd/4th -generation cephalosporin use^39^, while antimicrobial usage patterns in non-European settings represented in our isolate collection may differ. Such inter-country differences in antimicrobial use may influence baseline selection pressures and should be considered when interpreting host-associated resistance patterns^39,41^. This corresponds with low levels of resistance detected in European surveillance data which are obtained by culturing of samples for indicator bacteria on non-selective agar plates^41^. This context supports the interpretation that ceftiofur exposure can provide a strong selective advantage for ESBL-producing *E. coli* and, at least transiently, reduce competing susceptible *Enterobacteriaceae*. Consequently, these treatments likely effectively reduced non-resistant bacteria from the intestinal tract of the animals in this study, which were purchased from a commercial farm, and created favourable conditions for experimental bacterial colonization. The amoxicillin-treated animals also showed CFU counts higher than those of the control animals, but lower than those of the ceftiofur-treated group. Given the widespread use of amoxicillin since the 1960 s and the high levels of its associated resistance, its impact on intestinal bacteria would be expected to be less pronounced than that of ceftiofur. This assumption led us to include amoxicillin, a representative of the penicillin group of antibiotics, in addition to the cephalosporin ceftiofur, to create two levels of presumptive selective advantage in independent animal groups for the ESBL-producing experimental *E. coli* isolates assessed in this study.

Shedding of the inoculated isolates declined during the progression of the experiment, but many isolates were still detectable after enrichment, including, after approximately two months, in the second set of *post-mortem* examinations. This indicates persistence of resistant isolates, even in the absence of further antibiotic selection^43^ allowing recovery of the gut microbiota concomitant with fading out of the effects of the antibiotic compounds which had been administered on and flanking the inoculation day. This finding aligns with observations from EU AMR monitoring that selective plating yields a higher prevalence of ESBL-*E. coli* compared to non-selective culturing^42^, suggesting that minor ESBL-*E. coli* subpopulations may persist in the absence of antibiotic selection^44^. Importantly, enrichment increased detection sensitivity but precluded continued quantitative comparisons of CFU-based shedding patterns. *Post-mortem* sampling further indicated heterogeneity in isolate distribution within the gut: while isolates were predominantly regained from both tissue and content samples, a few isolates were instead exclusively detected in tissue (e.g., Cattle5/CET, Pig2/CET; Pig4/AMX), whereas others were only found in content samples (e.g., Human1/CET; Cattle7/CET), indicating varying colonization preferences and niche availability.

Studying host specificity using single isolate inoculations is laborious, time-, and resource-consuming. We, therefore, adopted a cocktail approach, simultaneously inoculating the animals with 17 different *E. coli* isolates, representing a collection of isolates from different hosts, host health status, country of origin, ST-based phylogeny and ESBL genes carried. This strategy effectively replicates the dynamic conditions found on pig farms, where animals frequently encounter multiple isolates of the same bacterial species simultaneously, owing to the practice of gathering pigs from nursing and farrowing farms in conventional finishing farms for rearing until market weight^45^. Isolate diversity is high during the initial weaner stage, reaching up to 13 different *E. coli* isolates in a pig^45^, similar to the maximum number of 12 experimental isolates detected here. Although multi-strain challenges can be influenced by bottlenecks and stochastic loss of minority variants^46,47^, we observed broadly consistent isolate profiles within treatment groups and time points, suggesting that stochastic effects did not dominate shedding patterns in this experiment. We note, however, that the experimental setting and animal age are not intended to replicate any single production scenario directly, and interpretation should focus on colonization dynamics under controlled conditions.

The most successful colonizers identified were Chicken1 (21225_2#112), Cattle1 (IMT38565), Pig3 (39533) and Generalist1 (21225_2#178), which showed the strongest and most persistent shedding, with Generalist1 exhibiting the highest overall detectability of all isolates applied in the cocktail. Other isolates predicted to be associated with the pig host, including Pig1 (IMT39234), Pig2 (IMT28138), and Pig4 (IMT38723), demonstrated colonization abilities from weak to intermediate across the treatment groups, while shedding of Pig5 (IMT38701) was not detected experimentally. Colonization performance did not correlate with acid resistance in the phenotypic screening performed here, suggesting that other traits (adherence, nutrient acquisition, competition, or plasmid burden) may contribute more strongly to persistence in this model.

A central aim of this study was to evaluate whether a phylogeny-based host-association framework aligns with in vivo colonization outcomes in pigs. Under the experimental conditions present here, predicted host-association based on phylogeny and isolation source did not significantly explain colonization capacity in pigs. In general, strong, intermediate and weak colonizers were distributed throughout the host preference groups, with no clear distribution for any host. This could be due to (i) host-association inferred from phylogenetic clustering and isolation host may not be sufficiently stringent or may be context-dependent, particularly within a species with strong population structure such as *E. coli*; (ii) because this study focused on ESBL-encoding isolates, the experimental set was enriched for lineages that are frequently recovered from multiple hosts and environments^48–51^, which may reduce detectable host restriction and/or (iii) that the pig host might be more or generally permissive to colonization by different *E. coli* isolates as indicated by the lack of identification of pig-host-specific genes, in contrast to cattle, chicken and human hosts in a genome-wide-association-study of the isolate collection used for selecting the experimental isolates^6^. Importantly, our data do not explicitly allow distinction between these explanations. Consequently, the proposed interpretations should be regarded as hypotheses rather than conclusions directly supported by the experimental results. Monitoring host colonization of the experimental isolates in other animal models will help to better evaluate the isolate classification method and colonization experiments with other isolate cocktails will help refine our understanding of host-association.

The strong colonizers, Chicken1 (ST1163), Cattle1 (ST362), Generalist1 (ST162), and Pig3 (ST410), belong to sequence types that are distributed globally and have been isolated across the One Health domains. Notably, Generalist1 and Pig3 share sequence types with globally distributed high-risk epidemic clones, as documented in previous studies^51,52^. Despite also belonging to international high-risk lineages, such as ST10, ST88, ST131, and ST648, other experimental isolates exhibited, in contrast, weak or intermediate colonization in pigs, indicating that global, widespread dissemination may not necessarily be associated with general colonization success as identified in this study, but that dissemination of different epidemic clones might be associated with different hosts.

Generalist1 and Pig3 harboured multiple resistance genes each. Chicken1 possessed 11 resistance genes, while Cattle1 possessed 13, surpassed only by Cattle2 and Pig2. Plasmids, including ESBL resistance plasmids, have been shown to contribute to bacterial survival within eukaryotic hosts^53,54^. If and which plasmid-encoded genes contribute to host colonization will require further experimental analysis.

*E. coli* isolates of ST162 have been previously isolated globally from various sources, but more commonly from poultry^51,55^. While the Generalist1 isolate was indeed isolated from poultry in Vietnam, the bioinformatic algorithm for host specificity scored it rather as a generalist. Its strong colonization of the pig GI tract suggests that the ST162 isolate is indeed able to successfully adapt to hosts other than birds. The first descriptions of ST410 *bla*_CTX−M−15_-encoding isolates, such as Pig3, were from samples collected from poultry, swine and cattle farms in Germany and Spain^56^. The *E. coli* ST410 lineage has evolved and now includes globally disseminated carbapenem-resistant clones, such as B4/H24RxC^57^. Within the ST410 lineage, genes encoding adherence and iron acquisition, such as *yadC*, *ybjI*, and *fhuA*, were acquired by this pandemic clone^57^, while *yadC* is missing and *fhuA* shows large sequence divergence in the Pig3 isolate. Low doses of an *E. coli* ST410 isolate persistently colonized and disseminated between broiler chicks in a small flock up to 35 days^58^, indicating strong colonization in another in vivo animal model. The remaining two strong-colonizing isolates, Chicken1 (ST1163) and Cattle1 (ST362), have been isolated from animals and humans, but have not yet raised attention in the literature implying that they have not developed into emerging high-risk isolates by now. To further our understanding of the variation of host adaptation within the species *E. coli*, beyond classical virulence factors like adhesins, it will be instrumental to identify the genetic elements and resulting metabolic phenotypes that contribute to colonization of the porcine gastrointestinal tract. The set of well-colonizing isolates identified here provides an experimental starting point for systematic analyses of genetic and phenotypic determinants contributing to colonization of the porcine gastrointestinal tract.

## Conclusion

Using a 17-isolate ESBL-*E. coli* cocktail, we established a reproducible in vivo pig model that resolved clear differences in isolate persistence and shedding, and showed that enrichment-based detection can uncover low-abundance colonizers not captured by non-enriched culture alone. Under the conditions tested, colonization success in pigs did not align with host-association inferred from phylogeny and isolation source, indicating that this classification approach alone is not predictive of colonization capacity in this host. Because the study focused on ESBL-encoding isolates and used a single host model, conclusions about host specificity are necessarily limited; additional host systems and isolate sets will be required to determine whether the mismatch reflects limitations of the classification framework, properties of ESBL lineages, host context, or a combination of these factors. At the same time, the cocktail-based approach enabled simultaneous evaluation of multiple isolates in vivo and proved useful for identifying isolates with strong colonization potential, providing a practical framework for future studies investigating bacterial host adaptation and colonization mechanisms.

## Materials and methods

### Isolate collection, host specificity scores and selection of test isolates

A collection of 1,198 *E. coli* isolates was assembled, collected in Germany, Spain, the United Kingdom and Vietnam between 2003 and 2018. The isolates’ original hosts were a mix of diseased and healthy cattle, chickens, humans, swine and wild boars^6^. For isolates obtained from diseased hosts, clinical syndromes recorded at sampling included swine diarrhoea and enteritis, bovine diarrhoea, bovine mastitis, bovine salmonellosis and human urinary tract infection.

A phylogeny-based bioinformatics approach was used as a classification method to predict the host specificity of the isolates. Briefly, isolates were assigned to a host-associated group if they were surrounded in the phylogeny trees by other isolates collected from the same host. To this end, 150 phylogenetic trees (bootstrap replicates) were generated using RAxML v8.2.4 (ACS_BINGAMMA model), based on a previously defined Roary pangenome matrix of all isolates^6^. For each tree and host species, a host specificity score was assigned, which represents the extent to which a particular isolate is surrounded by other isolates of a given host species. To quantify the extent to which an isolate belongs to the cluster of isolates sampled from the animal of origin, we first considered one sampled phylogenetic (binary) tree, exemplified in Fig. 1A. For a specific host species chosen (here chicken), one moves along the phylogenetic tree towards an individual leaf, representing an isolate). When a node in the phylogenetic tree is encountered (3/10), the fraction of chicken isolates is calculated for both subbranches. (for isolate SAP653, these are 0.222 (2/9) and 1 (1/1)). Then, the fraction of the subbranch containing the isolate of interest is divided by the sum of both subbranch fractions. In this case, this node-specific value *L*_*i*_ is 0.182 (0.222/(1 + 0.222)). This is repeated for all nodes (2/9, 2/3, 2/2), until the leaf isolate is reached. All *L*_*i*_ values are multiplied if more than one node is encountered. For isolate SAP653, this corresponds to four nodes (0.182 × 1 × 1 × 0.5 = 0.091). Then, values are corrected for leaf depth, by raising to the reciprocal of the number of nodes passed. In the example, this is 1/4, leading to *V*_*i*_ = 0.091^1/4^ = 0.549. *V*_*i*_ is, thus, a value between 0 (indicating that the isolate is not a chicken isolate), and 1 (all isolates in the branch leading to the isolate of choice were isolated from chickens). A value of *V*_*i*_ = 0.5 means that there is no specific tendency for the isolate to be surrounded by other chicken isolates (Fig. 1A). The calculations are repeated for each isolate and host for all 150 phylogenetic trees. For each isolate and host species, the geometric mean over the values per tree was used. The geometric mean was utilized to ensure that isolates that are surrounded by many other isolates of a host species in some trees, but not surrounded at all by isolates of the same host species in another tree do not get a high value. Only isolates which were consistently, i.e., in all trees, surrounded by isolates of the host species got a relatively high host specificity score.

The generalist score (i.e., indicative of an isolate associated with multiple host species) was calculated differently from the host-specific scores. For each of the four host species (human, chicken, cattle and pig), isolates were ranked according to their host-specificity scores. The geometric mean of these four ranks was then calculated for each isolate, producing the generalist score. This approach integrates host-association signals across multiple hosts, allowing isolates that show phylogenetic affinity to isolates from more than one host species to be classified as generalists, even if they were originally isolated from a single host.

Wild boar isolates were included in the pangenome phylogeny to represent the livestock–wildlife interface; however, they were not used to define host-associated clusters because their number and representation were insufficient to robustly establish wild-boar-associated groups under the criteria applied.

To select suitable isolates for the animal experiment, several criteria were applied: (I) Isolates belonged to *E. coli sensu stricto* (no cryptic clade isolates). (II) Isolates had either a relatively high host specificity score for a particular host species or were generalist isolates with no pronounced host specificity score across all hosts. (III) Isolates carried an ESBL gene. ESBL-positive isolates were prioritized due to their human/veterinary clinical relevance, their role as reservoirs of transferable AMR determinants and their global representation as indicator bacteria in AMR surveillance programs, such as EU monitoring or the WHO Tricycle. (IV) Isolates contained as few known virulence genes as possible, as identified through whole-genome sequencing (WGS) using tools such as VirulenceFinder. Preference was given to isolates from clinically healthy hosts to minimize confounding effects of virulence on the experiment. (V) Efforts were made to represent all sampled countries and time periods in the final list. However, due to collection composition, most isolates originated from Germany, with fewer isolates from other countries.

All isolates selected for the animal experiment were further characterized by antimicrobial susceptibility, acid resistance, and biocin/colicin production (Tables S2–S4; Supplemental Methods). Isolates were rendered rifampicin-resistant by serial passaging as previously described^29^, to enable selective recovery during the in vivo experiment.

### Preparation of the experimental bacteria (“cocktail”)

The bacterial cocktail was prepared for inoculation as described previously^29^. In brief, liquid cultures of all 17 isolates growing in the exponential phase were mixed at equal numbers (5.88 × 10^8^ cells per isolate) to reach a total of 10^10^ bacteria per inoculation dose. Culture mixtures were centrifuged (2,000 x g for 5 min, room temperature), the bacterial pellets resuspended in 10 ml of physiological saline containing 10% sodium bicarbonate to buffer stomach acid. After re-suspension, individual doses were immediately transported to the animal facility in ice coolers to inhibit bacterial growth.

### Animal experiment

Twenty-four clinically healthy female (*n* = 12) and surgically castrated male (*n* = 12) German Landrace pigs, 39–42 days old upon arrival, were obtained from a conventional commercial pig breeding herd in Germany.

The pigs originated from different litters within the same breeding herd. After weaning, animals were transported to the Friedrich-Loeffler-Institut (FLI), Isle of Riems, Greifswald, Germany, and housed in an environmentally controlled biological safety level 2 (BSL-2) animal facility. A three-week acclimatization period was implemented prior to experimental infection. Information on prior antimicrobial treatments or treatment rates within the source herd, and data on ceftiofur or amoxicillin usage at herd level, were not obtained. A detailed description on animal origin, housing, husbandry and acclimatization procedures can be found in the supplemental materials.

The experimental set-up was reviewed and approved by the local authority (State Office for Agriculture, Food Safety and Fisheries of Mecklenburg-Western Pomerania, Rostock, Germany, reference no. 7221.3-1.3.3–034/19). All procedures were conducted in accordance with the approved guidelines.

Animals were randomly divided into three groups of eight pigs each and housed in separate rooms in pens of 10.56 m². Animals received water ad libitum and were fed twice daily with age-appropriate commercial diets in restricted amounts according to body weight development. Environmental enrichment consisted of various types of rubber toys, jute ropes, and brushes for grooming, which were alternated regularly to promote exploratory behaviour. Bedding or rooting material was not provided, rubber mats were installed to enhance animal comfort.

On experimental days − 1, 0 and 1 (Figure S5), each group received a different treatment: group 1 (CET) was injected intramuscularly (i.m.) with 3 mg/kg of body weight ceftiofur per day (EXCENEL Flow^®^, Zoetis, Parsippany, NJ, USA); group 2 (AMX) received 15 mg/kg body weight amoxicillin i.m. per day (Duphamox^®^, Zoetis, Parsippany, NJ, USA). Both treatments were performed according to the summary of product characteristics.Group 3 (CON) received 2 ml of saline solution i.m. per day. Body weight used for dose calculation was recorded on day 0 (inoculation day) and was 12.5 kg (mean); final body weight at study end was 35 kg (mean).

Between experimental days − 1 and 0, the animals were fasted overnight but provided water *ad libitum*. On experimental day 0, the pigs were inoculated intragastrically with the *E. coli* cocktail using a gastric tube (B. Braun, Melsungen, Germany). Animals were previously sedated with azaperon (Stresnil^®^, Elanco, Greenfield, IN, USA) at 0.5 ml/20 kg body weight to reduce stress and enable precise inoculation. Following sedation and inoculation, the pigs quickly attained full consciousness and were fed immediately after full recovery. Post-inoculation, the pigs’ clinical condition was monitored once per day by observing overall general behaviour, in addition to food and water intake to assess animal well-being. Rectal swabs were collected from each animal on a daily basis from days 1–14 p.i., and every second day from day 15 until day 56 (end of the experiment), and processed for experimental bacteria colony-forming unit (CFU) count determination by plating on selective Gassner agar (Sifin, Berlin, Germany) and for DNA isolation.

Twelve animals, four per group, were euthanised for *post-mortem* examination on day 43 p.i. and the remaining 12 animals on day 56 p.i. The euthanasia of all pigs was performed using an overdose of Pentobarbital Sodium (Release, WDT, Garbsen, Germany). At *post-mortem* examination, the carcasses were opened to expose the intestinal tract. Samples were aseptically collected from four locations within the intestines: ileum, cecum, proximal and distal colon. For sampling, the intestinal portion was double-ligated at both ends with sterile thread (once in case of the cecum) to avoid shifting of contents. Sections were then separated from the rest of the intestinal tract by cutting between the ligatures to prevent content spilling. Once the section was separated, one end was opened and up to 50 ml of the content collected. Immediately after content collection, a large piece of intestinal tissue was removed, opened longitudinally to expose the mucosa and gently washed with tap water to remove any remaining content, without disturbing the mucus layer. A tissue piece of approximately 9 cm^2^ was then removed and immediately processed (1 gram of tissue was weighed, cut finely with a pair of scissors and added to 9 ml of CASO broth) for further bacterial detection.

### Bacteriologic examination of fecal swabs

Rectal swabs were collected, suspended in 1 ml of LB medium and allowed to rest at 37 °C for 30 min. The swab wash-offs were serially diluted from 10^− 1^ to 10^− 4^ and used for plate spotting on Gassner agar plates containing either no antibiotics, ceftiofur (4 µg/ml) or ceftiofur (4 µg/ml) and rifampicin (50 µg/ml). For spotting, 10 µl droplets of the dilutions 10^− 1^ to 10^− 4^ were gently deposited on each plate in duplicate and left open inside of a sterile flow cabinet for 1–2 min to allow drying of the droplet. After overnight incubation at 37 °C, colonies were counted in each droplet. In addition, 100 µl of the suspension from the rectal swabs were plated on Gassner agar plates containing ceftiofur (4 µg/ml) and rifampicin (50 µg/ml). After overnight incubation at 37 °C, plates were washed off using 2 ml LB, and the suspension used to isolate DNA with a commercial kit (peqGOLD Bacterial DNA Mini Kit, Peqlab, Erlangen, Germany). The leftover wash-off was mixed with 75% sterile glycerol in a 1:1 ratio, and immediately stored at −80 °C.

Once the CFU counts obtained from the rectal swabs had dropped to 1–2 colonies per plate, an enrichment step was added. For this, the rectal swabs were suspended in 1 ml of LB medium and allowed to rest at 37 °C for 30 min. These swab suspensions were serially diluted from 10^− 1^ to 10^− 4^ and used for spotting on Gassner agar plates without antibiotics for the total coliform count. Rifampicin was then added to the remaining suspension to a final concentration of 50 µg/ml, and all samples and plates were incubated overnight at 37 °C, aerobically. The enriched samples were then serially diluted from 10^− 1^ to 10^− 4^ and 10 µl droplets were spotted on Gassner agar plates containing ceftiofur (4 µg/ml) and rifampicin (50 µg/ml) and DNA harvested as described in the previous section.

### Bacteriologic examination of intestinal content

One gram each of liquid content or finely chopped tissue, as per what has been described above, were weighed and suspended in 9 ml of LB medium and allowed to rest at 37 °C for 30 min. One hundred µl of each suspension were serially diluted from 10^− 1^ to 10^− 4^ and used for plate spotting on Gassner agar plates as described in the previous section.

### Qualitative and quantitative detection by polymerase chain reaction targeting ORFan genes

The presence of the isolates was detected qualitatively and quantitatively by PCR with material derived from rectal contents. For qualitative PCR, DNA of the *E. coli* isolates obtained from the Gassner plates cultivated from the fecal swabs was used, representing the bacterial distribution throughout the experiment. For the qPCR assays, we utilized DNA directly isolated from intestinal contents collected during *post-mortem* examination, which reflects the numerical distribution of the bacterial population at the time of termination of the experiment.

ORFan gene identification, primer generation and specific PCR conditions for individual isolate identification were described previously^29^. Multiplex PCRs for the different host-associated groups of isolates (referred to as “multiplex-PCR” throughout the manuscript) were performed in a total volume of 25 µl per reaction, containing 2 µl purified DNA, 12.5 µl OneTaq 2x Master Mix with Standard Buffer (New England Biolabs Inc., Ipswich, MA, USA), 9.5 µl nuclease-free water and 0.5 µl each of 10 µM forward and reverse primers. PCRs were performed in a Biometra T3 Thermocycler System (Analytik Jena, Jena, Germany) using the conditions described in Table S5 in the supplemental material.

Quantitative PCR (qPCR) assays were also performed on the content samples obtained from different anatomical locations within the intestine at the *post-mortem* examination time-point of the experiment. The qPCR assay was designed to quantitatively identify which strong colonizing isolates (Generalist1, Pig3, Cattle1, Chicken1) were present at which intestinal site(s). For this, a CFX96 Touch Real-Time PCR Detection System (Bio-Rad Laboratories, Hercules, CA, USA) was used. Reactions contained a total volume of 20 µl, in which 2 µl purified DNA were used together with 10 µl Luna Universal qPCR Master Mix (New England Biolabs Inc., Ipswich, MA, USA), 7 µl nuclease-free water and 0.5 µl each of 10 µM forward and reverse primer. Each reaction was performed in triplicate. The cycling conditions included an initial denaturation step of 1 min at 95 °C followed by 40 cycles of 95 °C for 15 s and 60 °C for 30 s. As a housekeeping gene, *E. coli uidA* (beta-glucuronidase) was used^59^.

### Statistical analyses and mathematical modelling

#### Statistical analysis

Statistical data analysis was conducted in the R programming environment (v.4.2)^60^. Differences in the presence of isolates in the sample DNA, quantified by qPCR of their genomic DNA, between the antimicrobial-treated groups (amoxicillin or ceftiofur) and the control group were determined by Wilcoxon-Mann-Whitney tests (*p* ≤ 0.05) using the function wilcox.test^60^. Multiple adjustment of the p-values was performed using the p.adjust method (FDR). For each intestinal area (ileum, cecum, proximal colon and distal colon), individual statistics were generated to analyse area dependencies. Boxplots were generated to show distributions of the data using the ggplot2 package (v. 3.4.4)^61^.

#### Quantification of sampling uncertainty in coliform concentrations and ratios

In total, 600 fecal samples were obtained via rectal swabs (3 treatments x 8 pigs x 25 time points), each plated 16 times (2 treatments x 4 dilutions x 2 replicates), resulting in a total of 9,600 unique data points. Excluding 1,856 data points obtained after enrichment, the remaining 7,744 data points were all used to estimate per sample the most likely (rifampicin-)resistant and total coliform bacterial cell numbers, $\:{\widehat{C}}_{res}$ and $\:{\widehat{C}}_{tot}$ (CFU/sample), respectively. The loglikelihood function of the Poisson distribution with mean $\:{\mu\:}_{i}={V}_{i}\cdot\:C$ was maximized to estimate $\:{\widehat{C}}_{res}$ and $\:{\widehat{C}}_{tot}$. In this, *C* is the number of bacteria present in the undiluted sample, and *V*_*i*_ represents the volume plated per data point *i*, relative to the undiluted sample (i.e., the inverse of the dilution factor used in plating). Any plates where coliforms were too abundant to reliably count were incorporated into the loglikelihood function assuming a maximum countable threshold of 250 CFU per plate. 95% confidence intervals were computed based on the likelihood-ratio. When none of the *n* plates showed any coliforms, a one-sided 95% confidence interval was derived with the upper boundary at $\:-\mathrm{ln}\left(0.05\right)/\sum\:_{i=1}^{n}{V}_{i}$, i.e., based on the probability mass function for the Poisson distribution reduced at zero counts. Finally, the ratio between $\:{\widehat{C}}_{res}$ and $\:{\widehat{C}}_{tot}$, *r*_*res: tot*_, including 95% confidence interval, was estimated by combining the loglikelihood functions of both, with the constraint that $\:{\widehat{C}}_{res}\le\:{\widehat{C}}_{tot}$. Per time point, we drew 1,000 samples from the likelihood functions of *r*_*res: tot*_ for each individual pig. Per iteration, the draws of *r*_*res: tot*_ from all pigs in the same treatment group were averaged, before most likely (median) *r*_*res: tot*_ and the associated 95% confidence interval were derived.

#### Simulation of bacteria counts in rectal content

To reflect on the temporal trends of CFU ratios found in the rectal content of the animals, the pig digestive tract was simulated using a set of ordinary differential equations describing the transfer of inert particles (no growth or mortality) through its consecutive compartments:

1 $$\:\frac{d{N}_{stomach}}{dt}=-{k}_{stomach}\cdot\:{N}_{stomach}$$

2 $$\:\frac{d{N}_{s.int}}{dt}={k}_{stomach}\cdot\:{N}_{stomach}-{k}_{s.int}\cdot\:{N}_{s.int}$$

3 $$\:\frac{d{N}_{l.int}}{dt}={k}_{s.int}\cdot\:{N}_{s.int}-{k}_{l.int}\cdot\:{N}_{l.int}$$

4 $$\:\frac{d{N}_{exc}}{dt}={k}_{l.int}\cdot\:{N}_{l.int}$$

In these equations, *N* represents the number of inert particles in a specific compartment, denoted with their respective subscripts (*s.int* and *l.int* represent the small and large intestines, respectively; *exc* represents the amount cumulatively in rectal content), and *k* represents the transfer rates (hr^− 1^) through these compartments. These transfer rates were parameterised as the inverse of the mean retention times as reported by Wilfart^62^ for mature pigs. Simulations were adapted to the weight ranges attained during the experimental phase. Simulations were then run separately for liquid and solid particles, as transfer rates for the latter are slightly lower. Moreover, the rates in mature pigs (*k*_*adult*_) were allometrically scaled to reflect rates representative for younger animals (*k*_*piglet*_) as follows:

5 $$\:{k}_{piglet}={k}_{adult}\cdot\:{\left(\frac{{BW}_{piglet}}{{BW}_{adult}}\right)}^{ASF}$$

$$\:\frac{{V}_{gut,piglet}}{{{BW}_{piglet}}^{ASF}}=\frac{{V}_{gut,adult}}{{{BW}_{adult}}^{ASF}}$$

Allometric scaling factor (ASF) for gut volume = 1:

$$\:{V}_{gut,piglet}=\frac{{V}_{gut,adult}\cdot\:{{BW}_{piglet}}^{1}}{{{BW}_{adult}}^{1}}={V}_{gut,adult}\cdot\:{\left(\frac{{BW}_{piglet}}{{BW}_{adult}}\right)}^{1}$$

And similar for metabolic requirements but with an ASF of 0.75:

$$\:{met}_{piglet}={met}_{adult}\cdot\:{\left(\frac{{BW}_{piglet}}{{BW}_{adult}}\right)}^{0.75}$$

Now for transfer rates, scaling with the ratio of gut volume over metabolic requirements:

$$\:{k}_{piglet}={k}_{adult}\cdot\:\frac{{\left(\frac{{BW}_{piglet}}{{BW}_{adult}}\right)}^{0.75}}{{\left(\frac{{BW}_{piglet}}{{BW}_{adult}}\right)}^{1}}={k}_{adult}\cdot\:{\left(\frac{{BW}_{piglet}}{{BW}_{adult}}\right)}^{0.75}\cdot\:{\left(\frac{{BW}_{piglet}}{{BW}_{adult}}\right)}^{-1}={k}_{adult}\cdot\:{\left(\frac{{BW}_{piglet}}{{BW}_{adult}}\right)}^{0.75-1}={k}_{adult}\cdot\:{\left(\frac{{BW}_{piglet}}{{BW}_{adult}}\right)}^{-0.25}$$

In the formula, *BW*_*piglet*_ and *BW*_*adult*_ represented the average bodyweights of the study pigs (12.4 kg) and the mature pigs’ weight reported by^62^ (33 kg). The value of the allometric scaling factor (ASF; dimensionless) was based on the notion that transfer rates scale with the ratio of metabolic requirements over gut volume. Since gut volume scales isometrically with body weight, i.e., with an ASF of 1.00, and the interspecific scaling of metabolic requirements follows an ASF of 0.75 (Kleiber’s law), the ratio of the two scales with an ASF of −0.25. However, intra-, instead of interspecific scaling of metabolic requirements likely follows an ASF below 0.75, because older animals have more fat and require less maintenance energy per kg bodyweight. To capture this, temporal trends were simulated under ASF of −1.00 and − 0.25. All simulations were done with an initial inoculum (*N*_*stomach*_*[t0]*) of 10^10^ bacteria. To correctly represent the daily sampling, we extracted from the simulation outputs the 24-hour rolling cumulative sum of bacteria in the rectal contents.

#### Influence of antimicrobial treatment on composition of experimental isolate community shed

At 3–4 day intervals, rectal content samples from fecal swabs of individual pigs were subjected to ORFan multiplex-PCR to identify the presence or absence of each of the 17 donor isolates. This provides profiles of the experimental isolate community for each pig per time point. Using non-metric multidimensional scaling (NMDS), the relative influence of pig ID, antimicrobial treatment (ceftiofur, amoxicillin, control), and time of sampling on the (dis)similarity between these profiles was assessed using the *vegan* R package^63^. All samples, in which no experimental isolates were present, were excluded, because these provide no information on the community composition. NMDS was performed with number of dimensions *k* increasing from 2 to 10, each time with 15 tries (iterative) converging on the best solution based on the lowest stress. The lowest *k *below a threshold stress of 0.1 (good representation of actual dissimilarities^64^) was then selected. The Jaccard index was applied as distance metric, which is most suitable for presence-absence data^65^. Differences in profile similarity within and between treatment groups and sampling time points were assessed using ANOSIM (analysis of similarities).

### Predictive value of the phylogeny-based approach of genetic analysis to forecast relative colonization capabilities

To quantify colonization performance before enrichment-based detection, the area under the curve (AUC) was calculated for each isolate from the proportion of animals shedding that isolate over time. The approach focused on the percentage of animals within a treatment group shedding a specific isolate over the sampling days. The analysis window was defined as day 4 p.i. (inclusive) through day 13 p.i. (inclusive), reflecting the time period after simulated passage of non-interacting inert particles through the gastro-intestinal tract of pigs and before enrichment was introduced in the first group (see Fig. 2). To calculate the AUC, first, a linear interpolation (R function approx.^60^,) was used to generate comparable time courses. Afterwards, the AUC function of R package DescTools^66^ was applied with the trapezoid method. Isolates displaying an AUC of 0–5% of the maximum AUC value for the respective treatment group were defined as weak, isolates with an AUC value of 6–60% as intermediate and isolates with AUC values of 61–100% as strong colonizers. Boxplots were used to show differences in AUC ranges of the isolates in each treatment group using ggplot2, v. 3.4.4^61^. Statistical significances were computed using the unpaired two-samples Wilcoxon test.

## Supplementary Information

Below is the link to the electronic supplementary material.

Supplementary Material 1

## Data Availability

The data are available in the manuscript and its supplementary file. Additional data are available upon request. The sequences of the isolates are available under BioProject number PRJNA739205.
