# Supplementary material for: Intestinal colonization of weaner pigs by extended-spectrum-β-lactamase-encoding Escherichia coli classified for differential host-association using a phylogenetics-based approach
Source: Sci Rep. 2026 Jun 10;16:18020. doi: 10.1038/s41598-026-53668-0 (PMC13254097; doi:10.1038/s41598-026-53668-0)
Supplement: Supplementary file 1 — Supplementary Material 1 [file 41598_2026_53668_MOESM1_ESM.pdf]

Supplemental Materials

Additional strain classification information

To quantify the extent to which an isolate belongs to the cluster of isolates sampled from the animal of origin, we first considered one sampled phylogenetic (binary) tree. We labelled the leaves of the tree, which correspond to the isolates, “1” if the isolate was taken from the animal of origin, and “0” if the animal of origin was of a different species that the one of interest.

For each interior node of the phylogenetic tree, where a branch splits into two subbranches, the number of zeros and ones in both subbranches was calculated. This calculation considered: i) the total number of isolates for which the node is an ancestor, ii) the number of isolates of the respective host species for which the node is an ancestor, iii) the number of isolates within the branch that are phylogenetically related to the selected isolate of interest, and iv) the number of isolates of the particular host species within that branch.

The "selected isolate of interest" refers to isolates chosen based on their classification as either host-associated or generalist, as determined by prior *in silico* analysis (e.g., host-associated clustering or generalist behavior based on genomic data). Isolates are considered to belong to the same branch as the selected isolate if they share the same phylogenetic lineage, as inferred from the tree topology. These criteria allow for the examination of phylogenetic relationships and host-specific clustering, independent of phenotypic outcomes.

In our trees, all interior nodes have two subbranches. The degree of typicality for one of the host species (documented source of the sample) was calculated for both subbranches as follows: a branch is split into two subbranches with $n1$ and $n2$ leaves of which $c1$ and $c2$ leaves, respectively, are sampled from a given host species. If the fraction of leaves from a given host species is the same in both branches, i.e., $c1/n1=c2/n2$, both branches are similarly typical for the given host species, and the value should be close to ½ (0.5). If all leaves from a certain host species are in a single subbranch,

this subbranch will have a value close to 1. On the contrary, the value was closer to zero if there were low numbers of isolates of the host species present in the branch belonging to the selected isolate.

Next, all values for the subbranches corresponding to a leaf for all ancestral interior nodes, i.e., nodes from the leaf to the root were multiplied. The higher the value L_i for leaf *i*, the more leaf *i* falls in a part of the tree with many isolates sampled from the host species of interest. To correct for the fact that not all leaves have the same number of ancestral interior nodes, the k_i^{th} root of L_i, if leaf *i* has k_i ancestral internal nodes, was calculated using $V_i=(L_i)^{1/ki}$. To correct for the arbitrariness on how the value for each subbranch was calculated, a simplistic variant in which the value for subbranch 1 equals if $c_1+c_2>0$ and ½ (0.5) otherwise was used:

$$\frac{\frac{c_1}{n_1}}{\frac{c_1}{n_1}+\frac{c_2}{n_2}}$$

This implied that only leaves which were sampled from the animal of interest have a non-zero value V_i.

As different isolates may have had different numbers of ancestral nodes, the nth root of this product was calculated, where *n* is the maximum number of ancestral roots in all 150 phylogenetic trees. The nth root yielded a value per tree, per isolate and per host species. For each isolate and host species, the geometric mean over the values per tree was used. The geometric mean was interpreted as a relative measure which indicates to which extent the isolate was surrounded by isolates of the respective host species. The geometric mean was utilized to ensure that isolates that are surrounded by many other isolates of a host species in some trees, but not surrounded at all by isolates of the same host species in another tree do not get a high value. Only isolates which were consistently, i.e., in all trees, surrounded by isolates of the host species got a relatively high host specificity score. Also, if the selected isolate did not belong to the host species of interest, there was always an ancestral node of the aforementioned isolate at which the branch belonging to the selected isolate had no isolates of the host species while the other branch had. This implies that the selected isolate never got a very high geometric mean value. The generalist score (i.e. indicative of an isolate able to colonize all host

species) was determined by calculating the geometric mean of the host specificity scores for cattle, human, swine and chicken hosts.

Figure 1A provides an example for a small subbranch. For calculating chicken specificity, consider the formula given above for leaf 13219, which has values $c1$ = 1, $n1$ = 1 (a single leaf, isolated from chicken) and $c2$ = 2, $n2$ = 9 (two out of nine other leaves of the other subbranch isolated from chicken). Together, this gives an L_i of approximately 0.818. Together with the correction for leaf depth, V_i is $0.818^{1/1}$ = 0.818. Applying the formula for leaf SAP653, four nodes have to be considered. For the first node, the values are $c1$ = 2, $n1$ = 9, $c2$ = 1, $n2$ = 1, leading to an L_i of approximately 0.182. For the next node, the values for the formula are $c1$ = 2, $n1$ = 3, $c2$ = 0, $n2$ = 6, leading to an L_i of 1. The L_i of the next node is also 1, as there are again no chicken isolates in the alternative subbranch. The fourth node has values $c1$ = 1, $n1$ = 1, $c2$ = 1, $n2$ = 1 resulting in an L_i of 0.5. Multiplying all values of L_i for surpassed nodes leads to $0.182\times1\times1\times0.5 = 0.091$. As four nodes have been passed, the correction for leaf depth is $\frac{1}{4}$ leading to a V_i of $0.091^{0.25}$ = 0.549.

Experimental animals and housing conditions

Twenty-four clinically healthy female (n = 12) and surgically castrated male (n = 12) German Landrace pigs, 39–42 days old upon arrival, were obtained from a conventional commercial pig breeding herd in Germany (BHZP Garlitz, Langenheide, Germany). The herd operated under high biosecurity standards (personnel lock system, mandatory showering of staff, and routine vaccination programs). However, the animals were not provided with a specific-pathogen-free (SPF) status certificate.

The pigs originated from different litters within the same breeding herd. After weaning, animals were transported to the Friedrich-Loeffler-Institut, Isle of Riems, Greifswald, Germany, and housed in an environmentally controlled biological safety level 2 (BSL-2) animal facility. A three-week acclimatization period was intentionally implemented prior to experimental infection. This period allowed animals to adapt to the new environment, housing conditions, group composition, and animal care staff, and to recover from transport-associated stress, which is known to influence immune function. Only clinically healthy pigs were included in the study. While subclinical infections and inter-individual variation cannot be excluded in a conventional herd setting, the use of animals from different litters was considered representative of field conditions and may enhance generalizability of the results.

Information on prior antimicrobial treatments or treatment rates within the source herd, and data on ceftiofur or amoxicillin usage at herd level, were not obtained.

The experimental set-up was reviewed and approved by the local authority (State Office for Agriculture, Food Safety and Fisheries of Mecklenburg-Western Pomerania, Rostock, Germany, reference no. 7221.3-1-034/19). All procedures were conducted in accordance with the approved guidelines.

Animals were randomly divided into three groups of eight pigs each and housed in separate rooms in pens of 10.56 m². Animals received water ad libitum and were fed twice daily with age-appropriate commercial diets in restricted amounts according to body weight development. During

the first week after arrival, pigs received a mixture of Panto Start (17.5% crude protein; 13.8 MJ ME/kg) and Panto Fix (16.0% crude protein; 13.4 MJ ME/kg) for dietary adaptation, followed by Panto Fix until 12 weeks of age and subsequently Ceravis Schweinemast Rational (15.5% crude protein; 12.9 MJ ME/kg), with a one-week transition phase between diets. At 6–7 weeks of age, feed intake was approximately 700–850 g per pig per day and was gradually increased over time. Environmental enrichment consisted of rubber toys (floor-based and suspended), jute ropes, and brushes for grooming, which were alternated regularly to promote exploratory behaviour. Bedding or rooting material was not provided due to technical limitations of the wastewater treatment system in the BSL-2 facility; however, rubber mats were installed to enhance animal comfort.

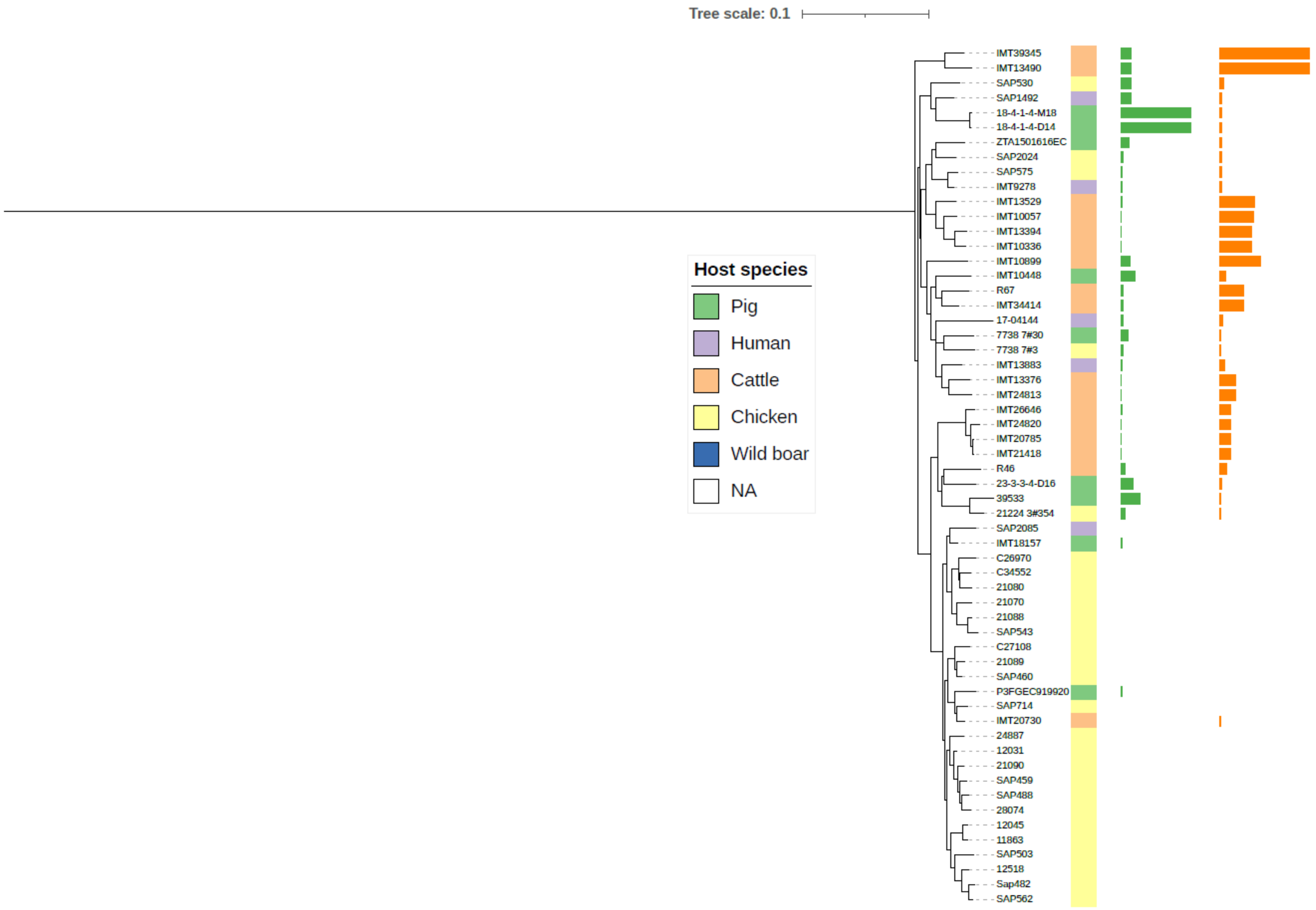

Figure S1. Close-up of the phylogeny tree branch containing the Pig3 and Cattle4 isolates

Table S1. ***E.coli*** **strain long list before further selection criteria was applied**

Strain ID	Host	Country of origin	Host Status	Multi-locus sequence types (ST, Achtmann[1])	Beta-Lactamase genes (*bla*)		
					CTX-M	OXA	TEM
**IMT10904**	Cattle	Germany	Diseased	ST399	$bla_{\text{CTX-M-1}}$		
**IMT10909**	Cattle	Germany	Diseased	ST448	$bla_{\text{CTX-M-1}}$		
**IMT13936**	Cattle	Germany	Diseased	ST10	$bla_{\text{CTX-M-1}}$		$bla_{\text{TEM-1B}}$
**IMT21418**	Cattle	Germany	Diseased	ST1615	$bla\text{C}_{\text{TX-M-1}}$		$bla_{\text{TEM-1B}}$
**IMT27294**	Cattle	Germany	Diseased	ST167	$bla_{\text{CTX-M-15}}$	$bla_{\text{OXA-1}}$	
**IMT28138**	Pig	Germany	Diseased	ST206	$bla_{\text{CTX-M-2}}$		$bla_{\text{TEM-1B}}$
**IMT34414**	Cattle	Germany	Diseased	ST88	$bla_{\text{CTX-M-1}}$		
**IMT34417**	Cattle	Germany	Diseased	ST1431	$bla_{\text{CTX-M-1}}$		$bla_{\text{TEM-1B}}$
**IMT38565**	Cattle	Germany	Diseased	ST362	$bla_{\text{CTX-M-1}}$		
**IMT38701**	Pig	Germany	Healthy	ST2067	$bla_{\text{CTX-M-1}}$		
**IMT38723**	Pig	Germany	Healthy	ST641	$bla_{\text{CTX-M-1}}$		
**IMT39234**	Pig	Germany	Diseased	ST361	$bla_{\text{CTX-M-15}}$		
**IMT39533**	Pig	Germany	Healthy	ST410	$bla_{\text{CTX-M-1}}$	$bla_{\text{OXA-1}}$	$bla_{\text{TEM-1B}}$
**IMT47016**	Chicken	UK	Diseased	ST117	$bla_{\text{CTX-M-1}}$		
**IMT47017**	Chicken	Vietnam	Healthy	ST1163	$bla_{\text{CTX-M-27}}$		$bla_{\text{TEM-1B}}$
**IMT47018**	Human	UK	Healthy	ST131	$bla_{\text{CTX-M-15}}$		
**IMT47019**	Human	UK	Diseased	ST131	$bla_{\text{CTX-M-15}}$		
**IMT47020**	Human	UK	Diseased	ST131	$bla_{\text{CTX-M-15}}$		
**IMT47021**	Human	UK	Diseased	ST636	$bla_{\text{CTX-M-15}}$		
**IMT47022**	Human	Vietnam	Diseased	ST1193	$bla_{\text{CTX-M-15}}$		$bla_{\text{TEM-1B}}$
**IMT47023**	Cattle	Germany	Healthy	ST361	$bla_{\text{CTX-M-15}}$	$bla_{\text{OXA-1}}$	$bla_{\text{TEM-1B}}$
**IMT47024**	Cattle	Germany	Healthy	-	$bla_{\text{CTX-M-15}}$		
**IMT47025**	Cattle	Germany	Diseased	-	$bla_{\text{CTX-M-15}}$		$bla_{\text{TEM-1B}}$
**IMT47026**	Pig	Germany	Healthy	-	$bla_{\text{CTX-M-15}}$	$bla_{\text{OXA-1}}$	$bla_{\text{TEM-1B}}$
**IMT47027**	Chicken	Spain	Unknown	ST1011			$bla_{\text{TEM-143}}$
**IMT47028**	Human	Germany	Diseased	-	$bla_{\text{CTX-M-1}}$		
**IMT47029**	Human	Germany	Healthy	ST301	$bla_{\text{CTX-M-1}}$		

**IMT47030**	Chicken	Vietnam	Healthy	-	*bla*$_{CTX-M-55}$	*bla*$_{TEM-1B}$

Table S2A – Resistome results (Plate 1)

id	Group	Antibiotics[1]							
		FFN	IMI	TIA	COL	NAL	STR	DOX	NEO
**21225_2#112**	Chicken_1	16	0.12	>=128	0.5	128	8	64	1
**SAP1847**	Human_1	8	0.12	>=128	0.5	>=256	8	1	1
**SAP1710**	Human_2	4	0.12	>=128	1	128	32	1	1
**IMT38565**	Cattle_1	256	0.12	>=128	0.5	>=256	4	4	0.5
**R45**	Cattle_2	4	0.12	>=128	0.25	>=256	16	16	0.5
**IMT13936**	Cattle_3	16	0.12	>=128	0.5	2	64	32	64
**IMT34414**	Cattle_4	4	0.12	>=128	0.5	2	8	2	0.5
**IMT10909**	Cattle_5	2	0.12	64	0.5	2	8	0.5	1
**9475_4#43**	Cattle_7	8	0.12	>=128	0.5	>=256	32	64	2
**IMT39234**	Pig_1	8	0.12	>=128	0.5	>=256	4	2	0.5
**IMT28138**	Pig_2	8	0.12	>=128	0.5	8	>=1024	64	>=128
**39533**	Pig_3	4	0.12	>=128	0.5	>=256	128	64	0.5
**IMT38723**	Pig_4	4	0.06	>=128	0.5	1	128	32	1
**IMT38701**	Pig_5	8	0.12	>=128	0.5	>=256	4	2	0.5
**21225_2#178**	Generalist_1	128	0.12	>=128	0.5	>=256	>=1024	16	2
**09-05726**	Generalist_3	4	0.25	>=128	0.5	2	8	2	2
**ZTA1601993EC**	Generalist_4	8	0.25	>=128	0.5	>=256	16	16	1

[1]FFN: Florfenicol; IMI: Imipenem; TIA: Tiamulin; COL: Colistin; NAL: Nalidixic acid; STR: Streptomycin; DOX: Doxycycline; NEO: Neomycin

Table S2B – Resistome results (Plate 2)

id	Group	Antibiotics[1]							
		CIP	GEN	ENRO	STX	MAR	TET	TIL	TUL
**21225_2#112**	Chicken_1	0,5	1	0,5	>=64/1216	0,5	128	>=256	>=64
**SAP1847**	Human_1	>=32	0,5	>=32	0,12/2,38	>=32	2	128	16
**SAP1710**	Human_2	0,25	0,5	0,25	2/38	0,5	1	64	16
**IMT38565**	Cattle_1	8	0,5	8	>=64/1216	8	32	64	4
**R45**	Cattle_2	>=32	32	>=32	2/38	16	128	128	32
**IMT13936**	Cattle_3	0,015	0,5	0,03	0,25/4,75	0,06	128	128	16
**IMT34414**	Cattle_4	0,015	0,5	0,03	0,12/2,38	0,03	2	128	16
**IMT10909**	Cattle_5	0,03	0,5	0,03	0,06/1,19	0,03	0,5	128	16
**9475_4#43**	Cattle_7	>=32	128	>=32	>=64/1216	16	256	>=256	>=64
**IMT39234**	Pig_1	>=32	0,5	>=32	0,06/1,19	16	1	128	16
**IMT28138**	Pig_2	1	32	1	0,12/2,38	1	256	128	>=64
**39533**	Pig_3	>=32	16	>=32	>=64/1216	16	256	128	16
**IMT38723**	Pig_4	0,015	0,5	0,03	0,06/1,19	0,03	256	128	16
**IMT38701**	Pig_5	8	0,25	16	0,06/1,19	8	1	128	32
**21225_2#178**	Generalist_1	8	128	16	>=64/1216	8	128	64	16
**09-05726**	Generalist_3	0,015	0,5	0,03	0,06/1,19	0,03	1	>=256	16
**ZTA1601993EC**	Generalist_4	8	64	16	>=64/1216	8	64	128	16

[1]CIP: Ciprofloxacin; GEN: Gentamicin; ENRO: Enrofloxacin; SXT: Trimethoprim-Sulfamethoxazole; MAR: Marbofloxacin; TET: Tetracycline; TIL: Tilmicosine; TUL: Tulathromycin.

Table S2C – Resistome results (Plate 3)

id	Group	Antibiotics[1]							
		AMP	AUG2	PEN	CEF	CEQ	CEP	FOT	FOP
**21225_2#112**	Chicken_1	>=128	4/2	>=64	>=128	>=64	>=256	>=64	>=64
**SAP1847**	Human_1	>=128	8/2	>=64	>=128	>=64	>=256	>=64	>=64
**SAP1710**	Human_2	>=128	4/2	>=64	>=128	>=64	>=256	>=64	>=64
**IMT38565**	Cattle_1	>=128	8/4	>=64	>=128	>=64	>=256	>=64	>=64
**R45**	Cattle_2	>=128	16/8	>=64	>=128	>=64	>=256	>=64	>=64
**IMT13936**	Cattle_3	>=128	16/8	>=64	>=128	>=64	>=256	>=64	>=64
**IMT34414**	Cattle_4	>=128	16/8	>=64	>=128	>=64	>=256	>=64	>=64
**IMT10909**	Cattle_5	>=128	16/8	>=64	>=128	>=64	>=256	>=64	>=64
**9475_4#43**	Cattle_7	>=128	8/4	>=64	>=128	>=64	>=256	>=64	>=64
**IMT39234**	Pig_1	>=128	8/4	>=64	>=128	>=64	>=256	>=64	>=64
**IMT28138**	Pig_2	>=128	16/8	>=64	>=128	>=64	>=256	>=64	>=64
**39533**	Pig_3	>=128	32/16	>=64	>=128	>=64	>=256	>=64	>=64
**IMT38723**	Pig_4	>=128	8/4	>=64	>=128	>=64	>=256	>=64	>=64
**IMT38701**	Pig_5	>=128	16/8	>=64	>=128	>=64	>=256	>=64	>=64
**21225_2#178**	Generalist_1	>=128	8/4	>=64	>=128	>=64	>=256	>=64	>=64
**09-05726**	Generalist_3	>=128	8/4	>=64	>=128	>=64	>=256	>=64	>=64
**ZTA1601993EC**	Generalist_4	64	4/2	>=64	0,25	0,06	8	0,12	0,5

[1]AMP: Ampicillin; AUG2: Amoxicillin-Clavulanic Acid; PEN: Penicillin; CEF: Ceftiofur; CEQ: Cefquinome; CEP: Cefalotin; FOT: Cefotaxime; FOP: Cefoperazone.

The experimental strains were comprehensively characterized for their phenotypic antimicrobial susceptibility profile by determining the minimal inhibitory concentration (MIC) of 24 substances belonging to eight antimicrobial classes (beta-lactams, fluoroquinolones, aminoglycosides, tetracyclines, phenicols, polymyxins, macrolides, and folic acid synthesis inhibitors (Tables 2a-c) by broth microdilution using custom-made microtiter plates (Sensitre, Zwalmen, The Netherlands). MIC values compared against the CLSI document VET01 (CLSI, 2018) with the control strain *E. coli* ATCC 25922 served as quality control strain.

Table S3. Test strain acid resistance testing results.

Strain ID	Group	pH 7,8				pH 2,5				pH 1,5	
		10^{-3}	10^{-4}	10^{-5}	10^{-6}	10^{-3}	10^{-4}	10^{-5}	10^{-6}	10^{-0}	10^{-1}
**21225_2#112**	Chicken cluster 1	TFTC	TFTC	TFTC	8	27	12	7	4,5	163	6
**SAP1847**	Human cluster 1	TFTC	TFTC	13	0,5	0,5	0	0	0	1	0
**SAP1710**	Human cluster 2	TFTC	TFTC	24,5	1	1	0,5	1	0	0	0
**IMT38565**	Cattle cluster 1	TFTC	TFTC	TFTC	TFTC	25,5	5,5	1,5	0	32	2
**R45**	Cattle cluster 2	TFTC	TFTC	TFTC	TFTC	TFTC	TFTC	TFTC	27	1120	97
**IMT13936**	Cattle cluster 3	TFTC	TFTC	30	2	8,5	1	0	0	0	0
**IMT34414**	Cattle cluster 4	TFTC	TFTC	54	11	0	0	0	0	0	0
**IMT10909**	Cattle cluster 5	TFTC	TFTC	71	13	TFTC	13	6	3,5	58	3
**9475_4#43**	Cattle cluster 7	TFTC	TFTC	22,5	2,5	TFTC	25	13	7	1	0
**IMT39234**	Pig cluster 1	TFTC	TFTC	19	1,5	TFTC	12,5	2	0	438	54
**IMT28138**	Pig cluster 2	TFTC	TFTC	52,5	4,5	69	13	0	1	237	16
**39533**	Pig cluster 3	TFTC	TFTC	27	3	TFTC	18,5	4	0,5	6	0
**IMT38723**	Pig cluster 4	TFTC	TFTC	TFTC	82,5	0	0	0	0	0	0
**IMT38701**	Pig cluster 5	TFTC	TFTC	TFTC	TFTC	90,5	40	23	9	128	2
**ZTA1601993EC**	Generalist cluster 4	TFTC	TFTC	15,5	3	38	17	35	22,5	0	0
**09-05726**	Generalist cluster 3	TFTC	64	13,5	2	0	0	0	0	0	0
**21225_2#178**	Generalist cluster 1	TFTC	TFTC	TFTC	22,5	TFTC	TFTC	28,5	8	380	40
**ATCC25922**	Chicken cluster 1	TFTC	59	7	1,5	5	2	0,5	0	2	0

Colony counts at pH 7,8, 2,5 and 1,5 and bacterial concentrations of 10^{-0} up to 10^{-6} depending on the pH (TFTC: Too full to count)

For acid resistance determination, all strains were grown overnight in 2 ml of sterile LB-medium at 37°C in a shaking incubator (180 rpm). After 16-20 hours, 10 µl of each overnight culture were inoculated in 1 ml of sterile LB with a pH adjusted to 1.5 (a), 2.5 (b) and 7.8 (c). Each tube was incubated for 2 hours at 37°C. After incubation, samples were plated on LB plates without antibiotics abiding the following pattern: a) twice, 100 µl of the samples at 10^0 and 10^{-1} were

plated; b) twice, 100 µl of the samples at 10^0 - 10^{-5} were plated; c) twice, 10 µl of the samples at 10^{-4} and 10^{-5} were plated. In addition, the initial overnight culture was also plated: twice, 10 µl of the samples at 10^{-5} - 10^{-7} were plated. Plates were allowed to incubate overnight at 37°C. The following day, all colonies were counted.

Table S4. Test strain colicin production results

Strain ID	Group	Inhibition
**21225_2#112**	Chicken cluster 1	Negative
**SAP1847**	Human cluster 1	Negative
**SAP1710**	Human cluster 2	Negative
**IMT38565**	Cattle cluster 1	Negative
**R45**	Cattle cluster 2	Negative
**IMT13936**	Cattle cluster 3	Negative
**IMT34414**	Cattle cluster 4	Negative
**IMT10909**	Cattle cluster 5	Negative
**9475_4#43**	Cattle cluster 7	Negative
**IMT39234**	Pig cluster 1	Negative
**IMT28138**	Pig cluster 2	Negative
**39533**	Pig cluster 3	Negative
**IMT38723**	Pig cluster 4	Negative
**IMT38701**	Pig cluster 5	Negative
**ZTA1601993EC**	Generalist cluster 4	Negative
**09-05726**	Generalist cluster 3	Negative
**21225_2#178**	Generalist cluster 1	Negative

[1]Growth of the test strains against a negative K12 strain (C600) on nutrient agar. Colicin production was measured by K12 growth inhibition (or crescentous spread of the colony on the agar).

For colicin production determination, one colony of each strain was individually incubated in 10 ml of nutrient broth (Tryptic Soy Broth) and incubated for 1 hour at 37 °C. Additionally an *E. coli* K12 strain with known absence of colicin production was grown using the same procedure, to be used as control strain. After the incubation period, Enterohemolysin agar (Sifin GmbH, Berlin, Germany) plates were used to test the strains. The *E. coli* K12 strain was streaked forming a vertical line in the middle of the plate using a swab, and allowed to dry shortly in a sterile environment. Four horizontal lines were then

made across the control *E. coli* K12 with each test strain, using a sterile swab, and allowed to dry. After plating, the plates were incubated at 37 °C overnight. For growth inhibition evaluation, the areas where the test strains and the control strain crossed were inspected and given a value (strong, middle and negative inhibition) based on the growth or lack of thereof around the control strain streak.

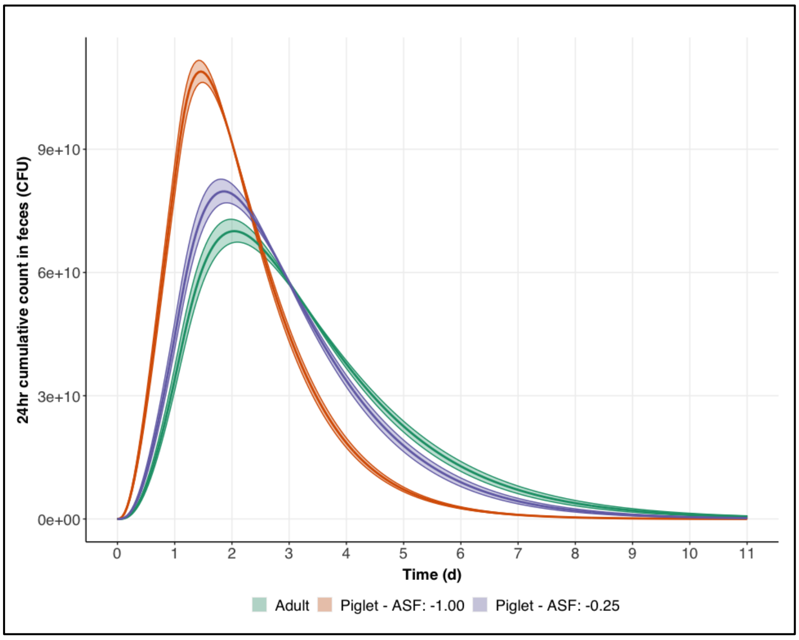

Figure S2. Simulated temporal trend of 24-hour cumulative CFU counts in pig feces, after inoculation with 1^{10} CFU bacteria. Bacteria were simulated as inert particles (no growth or mortality). Different temporal trends were obtained assuming different transfer rates through the digestive tract, representative for adult pigs, and for our piglets using allometric scaling factors (ASF) of -1.00 and -0.25. Lines represent average trends; shaded polygons represent ranges for liquid and solid particles.

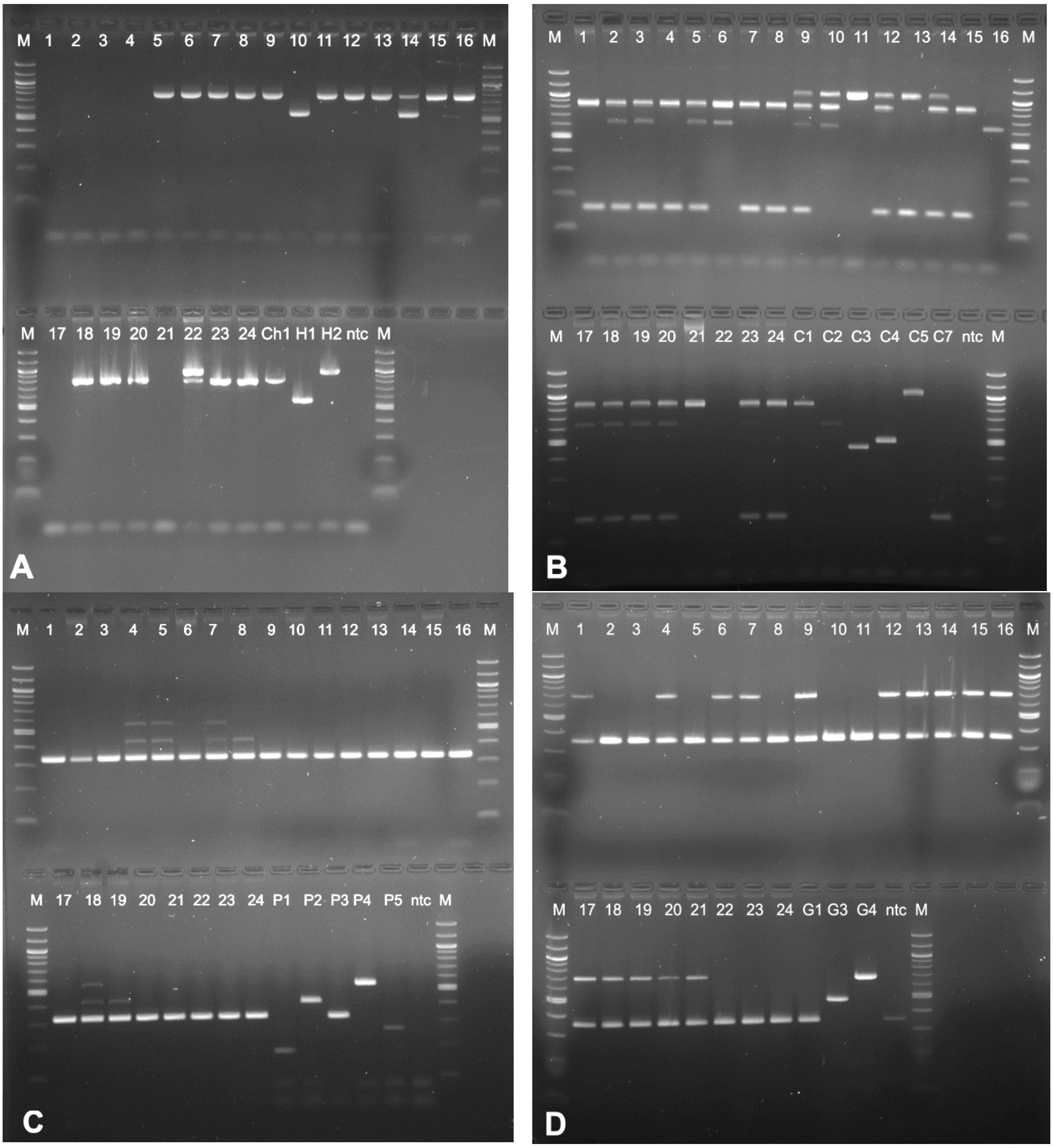

Figure S3: Isolate-specific Multiplex-PCRs for the detection of A – chicken and human isolates, B – cattle strains, C – pig strains and D – generalist isolates. The DNA used for this multiplex run was isolated on day 5 p.i.
Sample order in all gels - Upper row; M: Marker (100-bp); rows 1-8: CON animals; rows 9-16: AMX animals; M: marker (100-bp). Lower row: M: Marker (100bp); rows 17-24: CET animals; positive controls; non-template control (ntc); M: Marker (100-bp).
Postive controls - Ch1: Chicken1; H1: Human1; H2: Human2; C1: Cattle1; C2: Cattle2; C3: Cattle3; C4: Cattle4; C5: Cattle5; C7: Cattle7; P1: Pig1; P2: Pig2; P3: Pig3; P4: Pig4; P5: Pig5; G1: Generalist1; G3: Generalist3; G4: Generalist4.

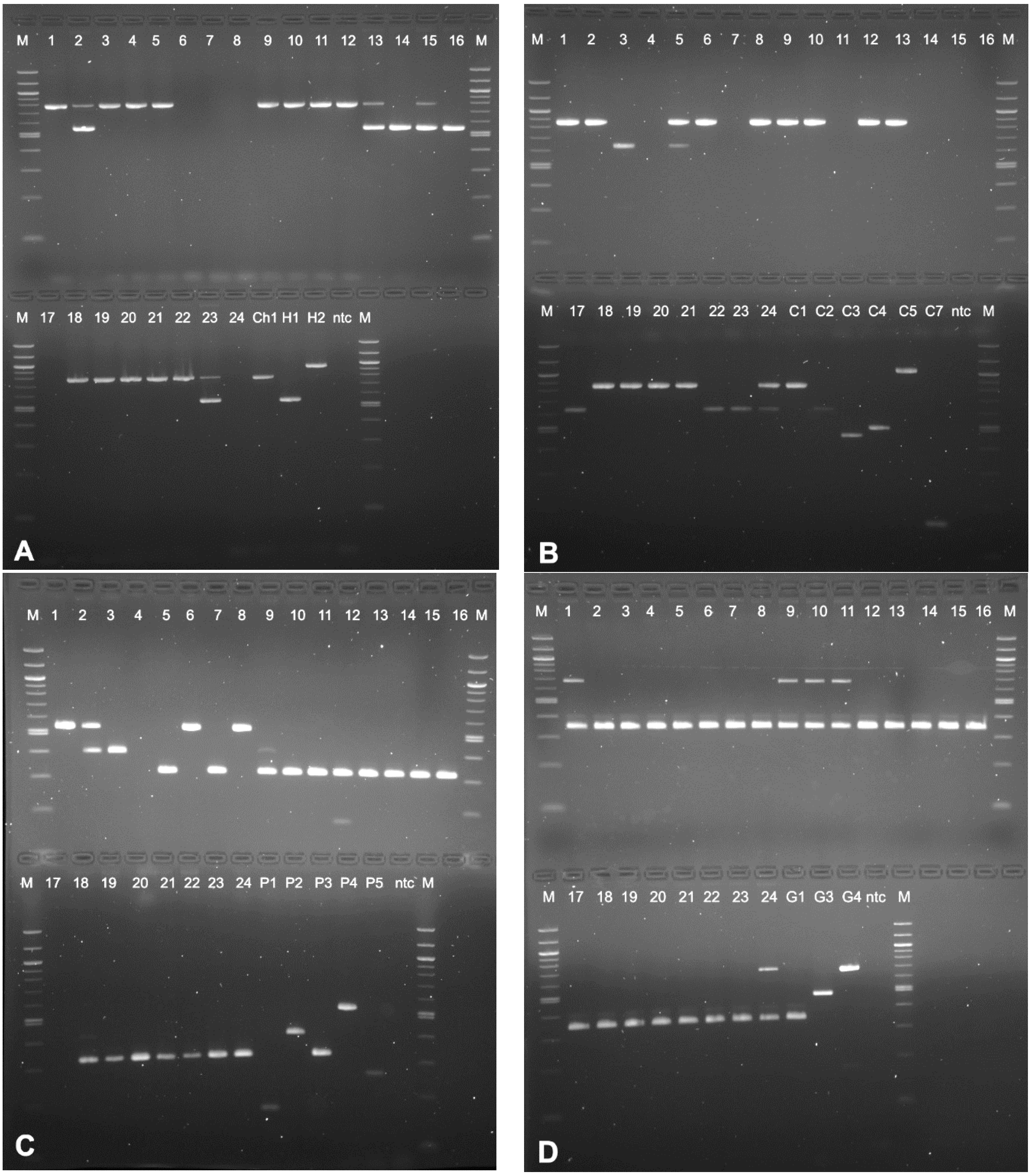

Figure S4: isolates-specific Multiplex-PCRs for the detection of A – chicken and human isolates, B – cattle isolates, C – pig isolates and D – generalist isolates. The DNA used for this multiplex run was isolated on day 25 p.i. when samples from all groups were already being subject to enrichment.

Sample order in all gels - Upper row; M: Marker (100-bp); rows 1-8: CON animals; rows 9-16: AMX animals; M: marker (100-bp). Lower row: M: Marker (100bp); rows 17-24: CET animals; positive controls; non-template control (ntc); M: Marker (100-bp).

Postive controls - Ch1: Chicken1; H1: Human1; H2: Human2; C1: Cattle1; C2: Cattle2; C3: Cattle3; C4: Cattle4; C5: Cattle5; C7: Cattle7; P1: Pig1; P2: Pig2; P3: Pig3; P4: Pig4; P5: Pig5; G1: Generalist1; G3: Generalist3; G4: Generalist4.

Table S5. Area under the curve values of isolate presence/absence during the peak shedding days

	Control group		Amoxicillin group		Ceftiofur group	
	AUC value [arbitrary units]	Classification[a]	AUC value [arbitrary units]	Classification[a]	AUC value [arbitrary units]	Classification
**Chicken1**	476.56	intermediate	1048.43	**strong**	1320.31	**strong**
**Human1**	29.68	weak	89.06	intermediate	135.93	intermediate
**Human2**	0.00	weak	0.00	weak	0.00	weak
**Cattle1**	868.75	**strong**	767.18	intermediate	1678.12	**strong**
**Cattle2**	282.81	intermediate	220.31	intermediate	660.93	intermediate
**Cattle3**	3.12	weak	1.56	weak	1.56	weak
**Cattle4**	0.00	weak	0.00	weak	0.00	weak
**Cattle5**	132.81	intermediate	178.12	intermediate	139.06	intermediate
**Cattle7**	278.12	intermediate	529.68	intermediate	628.12	intermediate
**Pig1**	84.37	intermediate	0.00	weak	220.31	intermediate
**Pig2**	409.37	intermediate	14.06	weak	167.18	intermediate
**Pig3**	617.18	**strong**	1398.43	**strong**	1817.18	**strong**
**Pig4**	285.93	intermediate	0.00	weak	35.9	weak
**Pig5**	0.00	weak	0.00	weak	0.00	weak
**Generalist1**	957.81	**strong**	1354.68	**strong**	1898.43	**strong**
**Generalist3**	0.00	weak	0.00	weak	0.00	weak
**Generalist4**	495.31	intermediate	1007.81	**strong**	471.87	intermediate

[a] classification rules applied were: “weak” = Isolates displaying an AUC of 0-5% of the maximum AUC value for the respective treatment group; “intermediate” = AUC value of 6-60%; “strong” = AUC values of 61-100%.

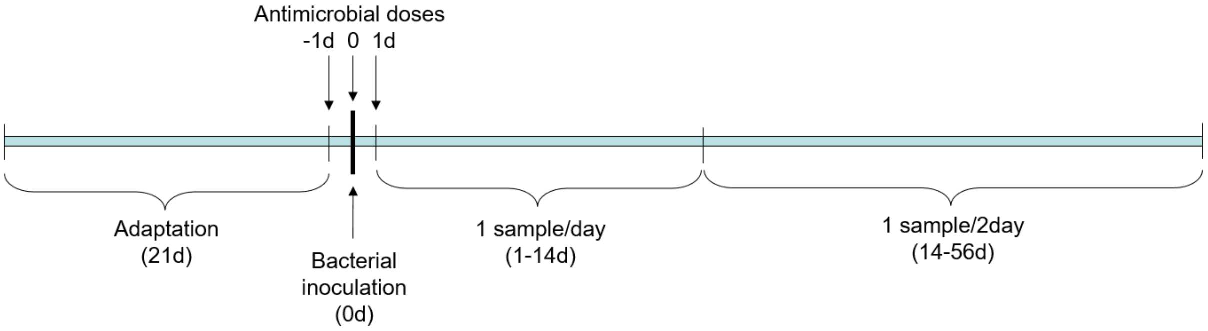

Figure S5. Experimental timeline. Upon arrival, the 24 animals were sorted in three groups with four males and four females per group and housed in three separate rooms. After a 21-day adaptation period, the AMX and CET groups were started on their respective antimicrobial treatments at days -1, 0 and 1, while the CON group was administered saline by injection. On day 0, all groups were inoculated with the 17-isolate cocktail.

Table S6. PCR conditions

Multiplex	Strains detected	Conditions		
		Step	T° (°C)	Time (s)
**Chicken-Human**	21225_2#112 (Chicken cluster 1)	Initial denaturation	94	30
	SAP1847 (Human cluster 1)	30 cycles	94	30
	SAP1710 (Human cluster 2)		48	40
			68	60
		Final extension	68	300
**Cattle**	IMT38565 (Cattle cluster 1)	Initial denaturation	94	30
	R45 (Cattle cluster 2)			
	IMT13936 (Cattle cluster 3)	30 cycles	94	30
	IMT34414 (Cattle cluster 4)		52	60
	IMT10909 (Cattle cluster 5)		68	70
	9475_4#43 (Cattle cluster 7)	Final extension	68	300
**Pig**	IMT39234 (Pig cluster 1)	Initial denaturation	94	30
	IMT28138 (Pig cluster 2)	30 cycles	94	30
	39533 (Pig cluster 3)		51	35
	IMT38723 (Pig cluster 4)		68	40
	IMT38701 (Pig cluster 5)	Final extension	68	300
**Generalist**	21225_2#178 (Generalist cluster 1)	Initial denaturation	94	30
		30 cycles	94	30
			49	40

	09-05726 (Generalist cluster 3)		68	45
	ZTA1601993EC (Generalist cluster 4)	Final extension	68	300
